# GRAPE: a pathway template method to characterize tissue-specific functionality from gene expression profiles

**DOI:** 10.1186/s12859-017-1711-z

**Published:** 2017-06-26

**Authors:** Michael I. Klein, David F. Stern, Hongyu Zhao

**Affiliations:** 1 Program in Computational Biology and Bioinformatics, Yale University, New Haven, CT USA; 2 Department of Pathology, Yale University, New Haven, CT USA; 3 Department of Biostatistics, Yale University, 60 College Street, P.O. Box 208034, New Haven, 06520-8034 CT USA

**Keywords:** Gene expression, Template, Relative expression analysis, Survival analysis, Personalized medicine, Cancer

## Abstract

**Background:**

Personalizing treatment regimes based on gene expression profiles of individual tumors will facilitate management of cancer. Although many methods have been developed to identify pathways perturbed in tumors, the results are often not generalizable across independent datasets due to the presence of platform/batch effects. There is a need to develop methods that are robust to platform/batch effects and able to identify perturbed pathways in individual samples.

**Results:**

We present Gene-Ranking Analysis of Pathway Expression (GRAPE) as a novel method to identify abnormal pathways in individual samples that is robust to platform/batch effects in gene expression profiles generated by multiple platforms. GRAPE first defines a template consisting of an ordered set of pathway genes to characterize the normative state of a pathway based on the relative rankings of gene expression levels across a set of reference samples. This template can be used to assess whether a sample conforms to or deviates from the typical behavior of the reference samples for this pathway. We demonstrate that GRAPE performs well versus existing methods in classifying tissue types within a single dataset, and that GRAPE achieves superior robustness and generalizability across different datasets. A powerful feature of GRAPE is the ability to represent individual gene expression profiles as a vector of pathways scores. We present applications to the analyses of breast cancer subtypes and different colonic diseases. We perform survival analysis of several TCGA subtypes and find that GRAPE pathway scores perform well in comparison to other methods.

**Conclusions:**

GRAPE templates offer a novel approach for summarizing the behavior of gene-sets across a collection of gene expression profiles. These templates offer superior robustness across distinct experimental batches compared to existing methods. GRAPE pathway scores enable identification of abnormal gene-set behavior in individual samples using a non-competitive approach that is fundamentally distinct from popular enrichment-based methods. GRAPE may be an appropriate tool for researchers seeking to identify individual samples displaying abnormal gene-set behavior as well as to explore differences in the consensus gene-set behavior of groups of samples. GRAPE is available in R for download at https://CRAN.R-project.org/package=GRAPE.

**Electronic supplementary material:**

The online version of this article (doi:10.1186/s12859-017-1711-z) contains supplementary material, which is available to authorized users.

## Background

One of the primary obstacles impeding the advancement of rational cancer treatments is the tremendous inter-tumoral heterogeneity. In some cancers there are well-established subtypes that account for a portion of the heterogeneity. However significant genetic and epigenetic variability remains within many of the subtypes and prevents reliable prediction of response to targeted treatments. In some cases the absence of targeted therapies is due to lack of therapeutically actionable mutational targets. For example in RAS driven cancers the protein product of the driver mutation itself is not directly druggable, and an understanding of which down-stream pathways are perturbed as a result of the driver mutation may help identify potential drug targets. Another example is the subset of triple-negative breast cancers that harbor PI3K mutations. Although inhibitors are available for these tumors, toxicity issues limit their usage. Finding secondary drug targets on a case-by-case basis can improve the therapeutic index for these patients via synergistic drug combinations. In these cases and many others, there is a critical need for computational methods that are capable of extracting functional information from the transcriptional profiles of individual samples.

Analysis of individual genes, e.g., using t-tests or fold changes to detect differentially expressed genes, is often unable to account for the complex interactions among genes whose protein products interact in complicated ways. Another problem with methods based on expression of individual genes is high correlations of expression within gene subsets, which muddles the identification of important ones in many circumstances. To overcome these limitations, researchers have developed many pathway-based methods in which the signals from pre-defined collections of genes, i.e. pathways, are considered together.

In this paper we present a method for inferring whether a pathway is differentially regulated based on the rankings of the genes using their expression values within the pathway. Our method is called GRAPE, an acronym for Gene-Ranking Analysis of Pathway Expression. GRAPE uses pairwise gene expression ordering within individual samples of a particular collection of genes to create a template representing the consensus ordering for components of the pathway within the collection. For every pair of genes (gene A, gene B), the template ordering is assigned to be either gene A > gene B or gene B > gene A depending on which ordering is present in the majority of samples in the collection.

The template concept behind GRAPE was inspired by Differential Rank Conservation (DIRAC) [1]. The difference between GRAPE and DIRAC is the way in which disagreement is quantified between a sample and a template, as well as between two templates. In DIRAC, the disagreement between a sample and a template is simply the proportion of reversals, i.e., gene pairs that are oppositely ranked in the sample compared to the template. This implicitly assigns an equal weight to all reversals. Instead, GRAPE uses a weighted penalty function in which the contribution of a reversal to the disagreement depends on the proportion of the reversals occurring within the reference collection. For example, consider a reversal in a new sample that is not part of the reference collection. If the reversal occurs in zero percent of the reference samples (i.e., unanimous vote) it will contribute much more highly to the distance between the sample and the template than if it had occurred in 40% of the reference samples. The purpose of this weighting function is to reduce the importance of gene pairs whose ordering is subject to high biological variability. In fact, DIRAC is a special case of GRAPE using a constant weight function. We hypothesize that by leveraging the flexibility afforded by the weight function to make efficient use of gene-ranking information, GRAPE may be ideally suited for the purpose of characterizing the tissue-specific behavior of individual pathways.

In the original description of DIRAC [1], the authors primarily used DIRAC to compare the amount of variability between different stages of cancer progression. An extension of DIRAC, Expression Variation Analysis, was developed to analyze gene expression variability within gene sets at improved computational efficiency [2]. This improvement was partially achieved by avoiding the use of templates when comparing the variability between phenotypes. Here, we show that GRAPE templates can be used in a much wider range of applications. For example, tissue-specific characterization of the typical pathway behavior and variability within healthy tissues may facilitate identification of perturbed pathways within individual tumor samples. We reason that the molecular underpinnings of a pathological state may be identified by detecting pathways exhibiting departure from the normal state. A similar idea has been previously applied at the level of individual transcripts in [3], where the authors used “anti-profiles”, i.e., the ranges of gene expression in normal samples, in an effort to diagnose colon cancer based on analysis of peripheral blood. We explore the value of applying this approach at the pathway-level using GRAPE.

We evaluate the usefulness of GRAPE in three domains. First we consider whether it is a viable tool for identification of tissue-specific pathway behavior. Next we consider the ability of GRAPE to integrate data generated from multiple distinct datasets within and between different technological platforms. This is an important consideration as both microarrays and RNA-Seq technologies are plagued by reproducibility issues, including dynamic range differences between platforms, gene-specific platform biases, batch effects, and poor resolution of lowly expressed genes [4–6]. Finally we consider an additional potentially powerful application of GRAPE by representing each tumor sample by a vector of pathway scores. We demonstrate how this pathway space representation can be used to analyze different disease subtypes. We further evaluate the ability of the pathway space representation to predict patient survival in several TCGA cancer subtypes.

## Methods

GRAPE is a generalization of the DIRAC method proposed in [1]. For completeness we describe the procedure from scratch.

### Binary representation of pathway gene expression

Consider a pathway consisting of m genes. We denote *P*=[*g*_1_,*g*_2_,…,*g*_*m*_] to be the expression levels of the genes belonging to the pathway within a particular sample. The continuous valued vector *P* is transformed into a binary valued vector *B* of length *m*∗(*m*−1)/2, corresponding to all unique pairs of distinct genes within *P*. If a pair of genes (*g*_*i*_,*g*_*j*_) are not equal, the value assigned is the indicator that *g*_*i*_ is less than *g*_*j*_. If *g*_*i*_=*g*_*j*_ the value is randomly assigned to be one or zero with probability 0.5. In practice the latter case happens almost exclusively when both genes are not expressed. The original representation of the sample *P*=[*g*_1_,*g*_2_,…,*g*_*m*_] thus becomes $B =[1_{g_{1}<g_{2}}, 1_{g_{1}<g_{3}}, \ldots, 1_{g_{m-1}<g_{m}}]$. The resulting representation contains identical information to ranking the *m* genes and resolving ties arbitrarily. The benefit of this binary representation is that information across samples can be aggregated by simple arithmetic operations, as discussed in the following sections.

### Construction of binary template and probability template over collection of samples

Consider a collection of samples comprising the reference phenotype. For a particular pathway, *P*, the probability template, denoted by *T*_*P*_, is constructed by averaging across each entry of the binary representation of all of the samples in the collection. That is, we have $\phantom {\dot {i}\!}T_{P} = [p_{g_{1}<g_{2}}, p_{g_{1}<g_{3}}, \ldots, p_{g_{m-1}<g_{m}}]$, where $p_{g_{i}<g_{j}}$ is the proportion of samples in the reference collection for which the expression of gene *i* is smaller than the expression of gene *j*. A binary template, denoted by *T*_*B*_, is then created by rounding the entries of *T*_*P*_ to zero or one. In the event that an entry in the probability templates is exactly 0.5 it is randomly assigned to be zero or one. *T*_*B*_ represents the consensus ordering within the reference collection, while *T*_*P*_ contains information about the amount of variability there is for each gene pair.

### Distance metric between a sample and a template

Construction of the binary template and probability template described above is identical between GRAPE and DIRAC. There is a crucial difference, however, in how the distance between a sample and a template is calculated. In DIRAC the distance is the proportion of violations between the sample and the binary template. Letting *E* be the set of all gene pairs, |*E*| denote the size of set *E*, we can write this as:

1 $$ D_{DIRAC} = \frac{1}{|E|} \sum_{(i,j)\in E} \left|T_{Bi,j} - B_{i,j}\right|.  $$

In GRAPE this calculation is modified to reduce the impact of uninformative gene pairs, i.e., gene pairs that appear in both orders with comparable frequency. To accomplish this a weight function is applied to the probability template:

2 $$ \begin{aligned} D_{GRAPE} = \frac{1}{S} \sum_{(i,j)\in E} w\left(T_{P,i,j}\right)\left|T_{Bi,j} - B_{i,j}\right|,\\ \text{where} \quad S = \sum_{(i,j)\in E} w(T_{P,i,j}). \end{aligned}  $$

For all of the results presented here, the following quadratic weight function was used:

3 $$ w(x) = (x - 0.5)^{2}, \forall x \in [0,1].  $$

In the supplementary information (Additional file 1: Topic S1) a procedure for optimizing the weight function based on the mean-variance ratio is presented. The quadratic weight function above was chosen because it displayed mean-variance characteristics that were very similar to the optimal weight function and the classification performances were not significantly different. Moreover, the simple analytical form enables much faster computation compared to the more time-consuming numerical optimization procedure.

### Pathway score function

The last step of GRAPE is to create a pathway score representing the degree of abnormal regulation of a particular sample relative to the reference collection. Given a collection of *n* reference samples, one first calculates *T*_*B*_ and *T*_*P*_ for a particular pathway. Then, for each sample in the reference collection, the GRAPE distance is calculated relative to the template. The median and inter-quartile distances of the distribution of distances are calculated and denoted *θ* and *δ*_0_, respectively. For a new sample, i.e., one that is not in the reference collection, we first calculate the distance from the template, *d*_*new*_, and then define the pathway score function, *f*, as follows:

4 $$ f(d_{new}) = \left\{\begin{array}{ll} 0 & \text{if } d_{new} \leq \theta \\ \frac{d_{new} - \theta}{\delta} & \text{otherwise} \end{array}\right.  $$

The purpose of this pathway score function is to quantify the abnormality of a pathway corrected for the center and spread of the distribution of distances of the reference samples for that pathway. To avoid situations where the inter-quartile distance is zero or near-zero, *δ* is set to be the maximum of *δ*_0_ and the distance of a single reversal of a pair that has template probability of 0.75. That is, *δ*=*max*(*δ*_0_,*w*(0.75)/*S*).

### Gene expression processing

In the case of genes with multiple mapped probes, the average over all mapped probes was used. We also repeated the inter-dataset analysis using max variance probe instead of the average for multi-probe aggregation, and we observed that the results did not change appreciably. For classification, all samples were standardized to have mean 0 and standard deviation 1 across all genes. This standardization only impacts the performance of the classification methods that are based on absolute expression, as GRAPE and DIRAC are invariant to any monotonic normalization. In the multi-dataset analyses, transcription profiles were considered to be only the genes that occur in every dataset within the analysis. Standardization was performed over the common set of genes, rather than all of the genes for each dataset.

All TCGA gene expression data were IlluminaHiSeq_RNASeqV2. All TCGA data were downloaded using the R package “TCGA2STAT”. Two samples were discarded from analysis due to suspicion of being outliers (see Additional file 1: Topic S2).

### Software details

Random Forests and SVM functions were taken from the R libraries “randomForest” and “e1071”, respectively. DIRAC was implemented without any external functions. GSEA was implemented using the desktop application [7]. Default parameters were used. Combat was implement using the R library “SVA” [8]. GSVA was implemented using the Bioconductor R package “GSVA” [9].

### Survival analysis

Multivariate survival prediction was performed using a penalized Cox Proportional Hazards Model (pCPHM) with a ridge (L2) penalty using the R library “penalized” [10]. Thirty iterations of 3-fold cross-validation were performed for each method. To reduce the noise in the comparison the same CV splits were used for each of the methods. Additionally, the cross-validation partitions were constrained to preserve the ratio of events to non-events within each fold. To match the number of features for GSVA and GRAPE, the set of GE features was filtered to include only the top 4500 features, ranked according to standard deviation. For each method, a two-step feature selection procedure was used within the training set. In the first step, univariate associations with survival were calculated for each feature using the function “gt” within the R library “globaltest” [11]. In the second step, the top *N* features having smallest *p*-value were chosen for the final model. A line search over the interval [5200] was used to identify the value for *N* for which the cross-validated likelihood is maximized. The function “optL2” in the “penalized” package was used to select the value of the ridge parameter lambda in the pCPHM, and to evaluate the internal cross-validated likelihood of the various sized models. All features were standardized in the pCPHM.

## Results

### Classification of healthy tissues using individual pathways within the same dataset

GRAPE templates were designed with the dual goals of sensitively capturing biological variation and being robust to noise. We evaluated these qualities by comparing the classification performance of GRAPE templates with four other methods: DIRAC, Pathway Centroid (PC), Support Vector Machine (SVM) and Random Forests (RF). In PC, the template is defined to be the centroid of the pathway genes and the distance of a sample to the template is the Euclidean distance. GRAPE, DIRAC and PC are examples of template generating methods, while SVM and RF are state of the art machine learning methods. PC is included to contrast the performance of a template based on relative gene expression with that of a template based on absolute gene expression. The term relative expression is used throughout this paper to mean methods that are based on gene rankings as opposed to the absolute expression values. This usage is adopted from [12], and refers to the relationships among the genes within a single profile, not to be confused with an alternative usage referring to the relationships between different profiles.

The Cancer Genome Atlas (TCGA) Pan-Cancer analysis project has made available a repository of genetic information from patient biopsies [13]. An underused subset of TCGA is the data from the matched normal sections of the biopsies that are often extracted along with the tumors. We analyzed four healthy tissue types from TCGA. Thirty-five RNA-Seq transcription profiles were randomly selected for each of lung, kidney, breast and head-neck healthy samples. Although more samples were available for lung, breast and kidney, a balanced dataset was used to better evaluate classification performance. The expression data from all samples were first standardized by subtracting the mean and dividing by the standard deviation. For each of the three template generating methods, a template for each tissue-type was first generated from the training set, and the samples in the testing set were then classified according to which of the templates they were most similar to using the relevant distance measure. For the two machine learning methods, classification was performed only using the genes within one pathway. In order to ensure robustness of the results, the classification accuracy was averaged over ten splits of five-fold cross validation.

The DIRAC and GRAPE shared template is comprised of two vectors: 1) a probability template indicating the proportion of samples for which each gene pair occurs in one of the two orderings (*T*_*P*_), and 2) a binary template derived by rounding the probability template to one or zero and representing the consensus order for the tissue type (*T*_*B*_). One of the advantages of DIRAC and GRAPE compared to the machine learning methods is that the templates can be easily visualized and interpreted. An example is shown in Additional file 1: Figure S1, where the binary templates (Additional file 1: Figure S1A) and probability templates (Additional file 1: Figure S1B) for each tissue type are shown for the KEGG “Folate Biosynthesis” pathway. This pathway only contains 11 genes and the corresponding templates consist of 55 gene pairs. The pairwise information in the binary templates corresponds to distinct consensus orderings of the genes for each tissue type (Additional file 1: Figure S1C). In DIRAC the distance from a sample to each template is the percentage of inversions relative to the binary template. In GRAPE each inversion is weighted by a function of the probability template value for the gene pair. The DIRAC five-fold cross validation accuracy is 0.82 and the GRAPE accuracy is 0.90 for this pathway.

**Fig. 1:**
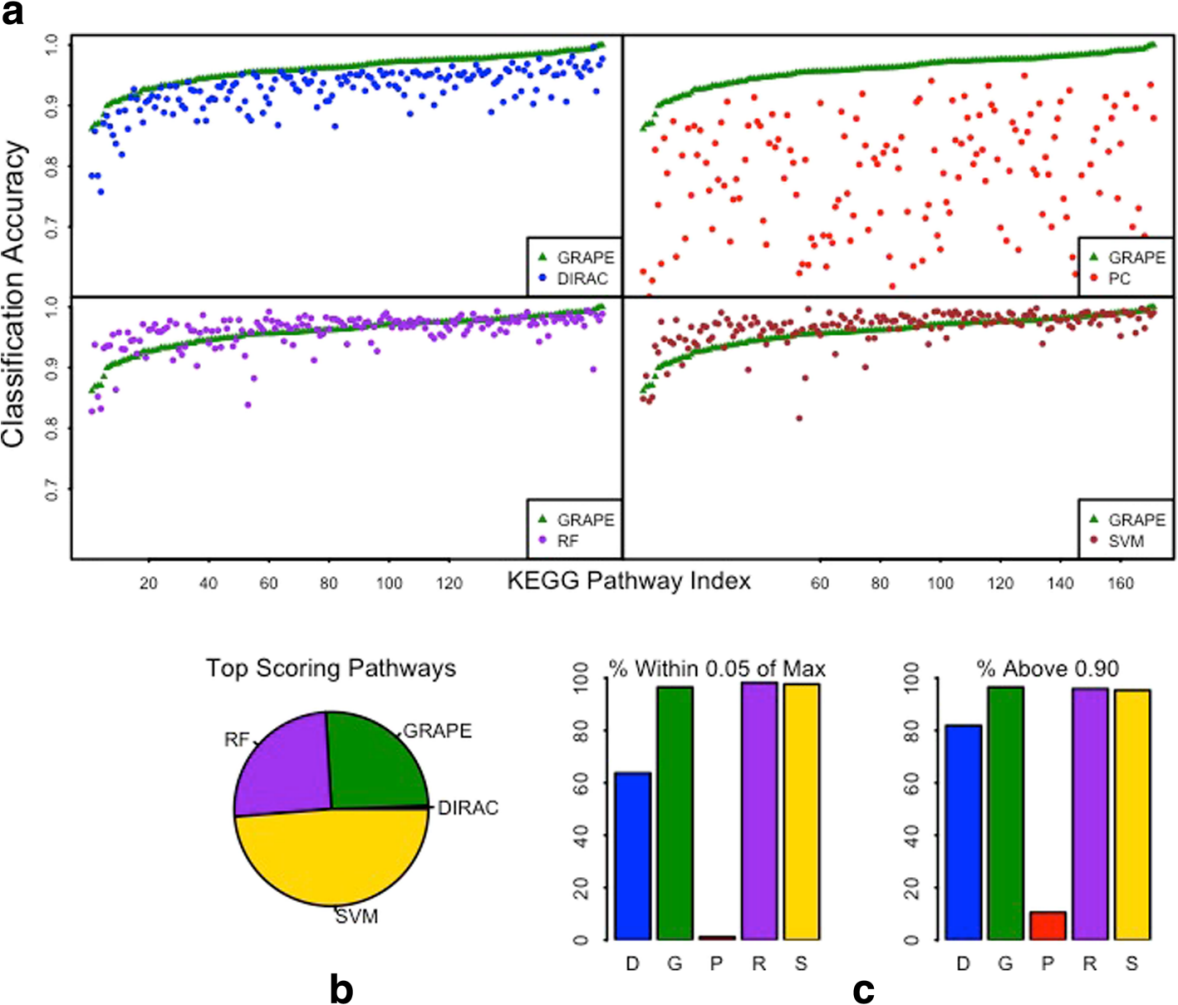
Classification of four different healthy tissue types within TCGA dataset: breast, lung, head and neck, and kidney. Five-fold cross-validation was performed 10 times for 171 KEGG and BioCarta pathways using each of five methods. In each panel, the pathways are ordered according to the classification performance of GRAPE (*green*). The viewing range is lower bounded by 0.6, as only PC had any pathways (n=15) that failed to achieve this threshold. **b** Proportion of pathways for which each method had largest accuracy. If multiple methods achieved the max (within.001 counted), the credit for the pathway was fractionated. **c** Proportion of pathways that achieved classification accuracy within 0.05 of the best performance (*left*) and proportion that achieve at least 0.90 classification accuracy (*right*)

The classification accuracy of all five methods was compared for the set of KEGG and BioCarta pathways that have between 25 and 100 genes (Fig. 1). This lower bound on the pathway size was chosen because we observed that GRAPE and DIRAC classification accuracies were strongly correlated with pathway length for pathways with fewer than 25 genes (Additional file 1: Figure S2). Although both GRAPE (Fig. 1 a, green) and DIRAC (Fig. 1 a, blue) achieve greater than 85% accuracy for the majority of pathways, GRAPE significantly outperforms DIRAC in this context. This indicates that the addition of a weight function to decrease the impact of noisy gene pairs improves the discriminative power of the GRAPE algorithm. Both GRAPE and DIRAC dramatically outperform PC (Fig. 1 a, red). This suggests that it is advantageous to use rank-based template methods over absolute gene expression values, which are subject to much greater variability. Among the three template-generating methods, GRAPE achieves the highest classification accuracy in 169 out of 171 pathways. In this context, GRAPE was comparable to RF (Fig. 1 a, purple) and SVM (Fig. 1 a, brown) in classification performance. GRAPE achieves a higher classification performance than RF in 79 out of 171 pathways, and it outperforms SVM in 57 out of 171 pathways. The results for the full set of KEGG and BioCarta pathways (max 250 genes) are summarized in Additional file 1: Table S1.

SVM achieves the best classification performance for the largest percentage of pathways (49%), followed by GRAPE (25%), RF (25%), and DIRAC (1%) (Fig. 1 b). DIRAC achieves classification accuracy within 0.05 of the top performing method in 64% of the pathways, compared to 96% for GRAPE, 1% for PC, 98% for both RF and SVM (Fig. 1 c, left). Out of 171 pathways, 165 pathways achieved at least 90% classification accuracy using GRAPE (Fig. 1 c, right), and 123 pathways achieved classification accuracy of at least 95% (not shown). For SVM and RF, 164 and 163 pathways achieved 90%, respectively, and 134 and 142 pathways achieved 95%, respectively. For DIRAC, 140 and 55 pathways achieved classification accuracies of 90% and 95%, respectively. By far the poorest performing method was PC, for which only 18 pathways achieved at least 90% classification accuracy and none achieved 95%.

### Classification across multiple datasets

One of the challenges of microarray-based gene expression profiling is lack of reproducibility between distinct experiments of similar biological samples [4, 5]. Part of the problem is the use of numerous distinct platforms for generating gene expression data. However, lack of concordance of gene expression often persists even among experiments containing similar or even identical biological samples using the same microarray platform [5]. We hypothesized that pathway methods based on relative expression may be more robust to experimental inconsistencies than methods based on absolute expression. We reasoned that relative expression methods may be more robust to discrepancies in the range of expression values that often exist among distinct datasets [5]. To evaluate this hypothesis, we compiled a collection of 11 datasets of three normal tissue types: muscle, lung and colon (Table 1). Six of the datasets were generated using Affymetrix Human Genome U133 Plus 2.0 Array (GEO accession: GPL570). Among these six datasets, two datasets were chosen for each tissue type. The remaining five datasets were generated using Agilent-014850 Whole Human Genome Microarray 4x44K G4112F (GEO accession: GPL6480). These consisted of two muscle datasets, two lung datasets and one colon dataset. These datasets were selected by manually querying GEO for datasets with at least 15 samples of the appropriate healthy tissue. Only healthy samples were included except for the colon datasets, where we did include samples from patients with inflammatory bowel disease (IBD) and low-grade dysplasia because of insufficient quantity of healthy samples.

**Table 1:** Gene Expression datasets used in healthy tissue classification

GEO accession	Platform	Tissue	# Samples	Figs. ^*a*^
GSE47881	Affymetrix (GPL570)	Muscle	45	2, S4
GSE9419	Affymetrix	Muscle	22	2
GSE4302	Affymetrix	Lung	44	2, S4
GSE5058	Affymetrix	Lung	24	2
GSE4183 ^*b*^	Affymetrix	Colon	23	2, S4
GSE9254	Affymetrix	Colon	19	2
GSE42507	Agilent (GPL6480)	Muscle	44	S3, S4
GSE23697	Agilent	Muscle	35	S3
GSE40588	Agilent	Lung	60	S3, S4
GSE15197	Agilent	Lung	39	S3
GSE41667 ^*c*^	Agilent	Colon	33	S4

^a^Indicates which classification analyses each dataset was part of. The classification analyses are indexed by figures, with Fig. 2 referring to the Affymetrix only multi-dataset analysis, Additional file 1: Figure S3 referring to the Agilent only multi-dataset analysis and Additional file 1: Figure S4 referring to the mixed-platform analysis

^b^8 healthy, 15 IBD

^c^12 healthy, 21 low grade dysplasia

**Fig. 2:**
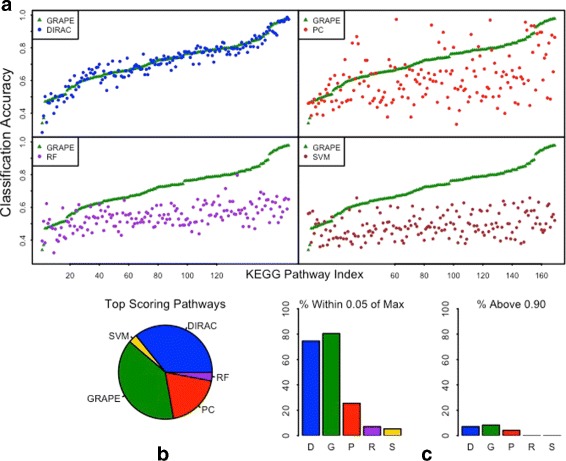
Multiple dataset classification of tissue types. **a** Classification across 6 Affymetrix (GPL570) datasets: 2 lung, 2 muscle, and 2 colon (Table 1). Nineteen samples were randomly selected from each of the six datasets. There are eight possible ways to split the datasets such that each tissue type is contained in each of the training and testing sets. The score for each pathway is the average of all eight splits. **b** and **c**: as in Fig. 1

Figure 2 shows the classification performance of the five methods when distinct Affymetrix datasets were used for training and testing. There are eight possible ways to split the six datasets such that one dataset from each of the three tissue types is contained in each of the training and testing sets, respectively. The classification accuracies were calculated to be the average of all eight splits. Two pathways were removed because they had fewer than 25 genes covered by the Affymetrix array. All five methods had lower classification accuracies in this setting in comparison to analyses of single datasets. The GRAPE average classification accuracy across all pathways was 0.96 for a single dataset and 0.72 with multiple datasets. The DIRAC and PC average classification accuracies showed a similar decrease, from 0.92 to 0.71 for DIRAC and from 0.75 to 0.60 for PC. The fall-off was much greater for SVM and RF, as the average classification accuracy decreased from 0.96 to 0.49 for SVM and from 0.96 to 0.54 for RF. The numbers of pathways achieving classification accuracies greater than 90% (Fig. 2 c) were 14 for GRAPE (9 achieved ≥ 95%), 12 for DIRAC (8 achieved ≥ 95%), 7 for PC (3 achieved ≥ 95%) and zero for both RF and SVM.

In order to investigate if this pattern is specific to Affymetrix datasets, we repeated the analysis using four Agilent datasets, two for lung and two for muscle (Table 1, Additional file 1: Figure S3). Higher classification performances were expected compared to the Affymetrix experiment, since only two different tissue types were used in the Agilent experiment. The average classification accuracies over all 169 eligible pathways were 0.92 for DIRAC, 0.91 for GRAPE, 0.74 for PC, 0.65 for RF and 0.62 for SVM. The numbers of pathways achieving classification accuracies greater than 90% (Additional file 1: Figure S3C, right) were 114 for GRAPE (98 achieved ≥ 95%), 116 for DIRAC (98 achieved ≥ 95%), 41 for PC (35 achieved ≥ 95%), 12 for RF (9 achieved ≥ 95%) and zero for SVM.

We next evaluated classification involving multiple datasets from two microarray platforms (Additional file 1: Figure S4). One Affymetrix dataset for each of muscle, lung and colon was combined with one Agilent dataset for the same tissues (Table 1). The sizes of the datasets were balanced by randomly selecting 23 samples from each dataset. The classification accuracies were calculated by averaging over all eight possible splits of test and train sets, as was done for Fig. 2. The classification performances for all five methods were dramatically lower in this case in comparison to the single platform cases. The average classification performance was 0.42 for both GRAPE and DIRAC, 0.48 for PC, 0.44 for RF and 0.47 for SVM. None of the methods had any pathways achieving classification accuracies of 90%, and only PC had any pathways (n = 3) achieving 80% accuracy.

All three multi-dataset classification experiments were also performed using the full set of KEGG and BioCarta pathways (max 250 genes) and the results are summarized in Additional file 1: Table S1.

### Rates of absolute and relative inter-dataset differential expression

We hypothesized that the superior classification performance of the relative expression methods across datasets of the same microarray platforms (Fig. 2, Additional file 1: Figure S3) is caused by differences in the rates of inter-dataset differential expression between relative expression and absolute expression methods. We defined the absolute rate of differential expression (ARDE) for a pair of datasets to be the proportion of genes for which the nominal *p*-value is below 0.01 using the two-sided t-test. We defined the relative rate of differential expression (RRDE) to be the proportion of gene-pairs for which the exact two proportion binomial test yields nominal *p*-values below 0.01. We considered the set of genes that occur in at least one KEGG or BioCarta pathway (only pathways with ≤ 150 genes were considered). This set contains 4470 distinct genes that appear in the Agilent datasets and 4413 genes that appear in the Affymetrix datasets. To reduce the computational cost, the RRDE was estimated by averaging the RRDE over 10 random draws of 200 genes. The variation across the draws was minimal, lending confidence to the accuracy of the estimation procedure. The ARDE was calculated over the full set of genes using both standardized (ARDE-S) and non-standardized (ARDE-N) versions of each dataset, where standardization was applied to the full gene expression profiles.

The RRDE, ARDE-N and ARDE-S were calculated for each pair of datasets used in the Affymetrix analysis (Additional file 1: Table S2). The average ARDE-S across the three homo-tissue dataset pairs was 0.78 (ARDE-N = 0.97) and the average ARDE-S across the nine hetero-tissue dataset pairs was 0.83 (ARDE-N = 0.93). The corresponding average RRDEs for the homo- and hetero-tissue dataset pairs were 0.26 and 0.37, respectively. The hetero-tissue average rate of differential expression is expected to encompass both the biological differences among the tissues and also the batch effects among the different experiments. By contrast, the corresponding homo-tissue rates are a reflection of the magnitude of the batch effects alone, as true biological differences are not expected between different datasets of the same disease-free tissue. Therefore the ratio of the hetero-tissue to homo-tissue average rate of differential expression can be considered a measure of the signal to noise for each metric of differential expression rate. For ARDE-S this ratio was 1.07 (0.96 for ARDE-N), and for RRDE this ratio was 1.39 (Table 2). A similar pattern was observed among the four Agilent datasets, for which the ARDE-S, ARDE-N and RRDE ratios were 1.09, 1.12 and 1.63, respectively (Table 2, Additional file 1: Table S3). This supports the observation from the classification analyses that biological signal can be more readily separated from batch-effects using relative expression methods rather than absolute expression methods. Further support for the relationship between the hetero-tissue/homo-tissue ratio and classification performance was observed when considering the datasets from mixed Affymetrix/Agilent analysis. In this case the ratios were 1.01, 1.02 and 1.14 for ARDE-S, ARDE-N and RRDE, respectively (Table 2, Additional file 1: Table S4).

**Table 2:** ARDE and RRDE of Affymetrix Datasets

	ARDE-N	ARDE-S	RRDE
Affymetrix Mean Homo-Tissue	0.97	0.78	0.26
Affymetrix Mean Hetero-Tissue	0.93	0.83	0.37
***Affymetrix Hetero/Homo Ratio***	**0.96**	**1.07**	**1.39**
Agilent Mean Homo-Tissue	0.86	0.84	0.22
Agilent Mean Hetero-Tissue	0.96	0.92	0.36
***Agilent Hetero/Homo Ratio***	**1.12**	**1.09**	**1.63**
Mixed Mean Homo-Tissue	0.95	0.8	0.48
Mixed Mean Hetero-Tissue	0.97	0.81	0.54
***Mixed Hetero/Homo Ratio***	**1.02**	**1.01**	**1.14**

Ratios of hetero/homo tissue differential expression rates emphasized for each experimental setting

We evaluated how the relative differential expression pairs are distributed among the genes. For this analysis we compared the two Affymetrix muscle datasets and the two Affymetrix lung datasets. The full set of 4413 pathway genes were considered for this analysis. We define the Differential Ordering Measure (DOM) of a gene to be the number of significant differentially ordered pairs (exact two proportion binomial test P ≤0.01) containing the gene, divided by the average number of significant differentially ordered pairs over all of the genes. Additional file 1: Figure S5A shows the distributions of DOMs in the muscle and lung comparisons. Both distributions have long right tails, indicating that a small subset of genes are accounting for a large proportion of batch effects. To further visualize the impact of the genes with largest DOMs, we plotted the cumulative contribution of all of the genes ordered from largest to smallest DOM (Additional file 1: Figure S5B). Additional file 1: Table S5 contains the top 100 differentially ranked genes in each comparison and their corresponding DOMs. Inconsistent gene pairs did not exclusively occur among genes expressed at low levels, as some genes were observed to be among the most expressed in one dataset and among the least expressed in the other dataset.

### Comparison with combat

One of the most popular methods for removing batch effects in gene expression data is Combat [8]. We applied Combat to each of the multi-data settings described above (Figure 2, Additional file 1: Figure S3 and S4) and then calculated the classification accuracies of RF and SVM after batch correction. For example, in the Affymetrix 3 tissues experiment, Combat was applied over all six Affymetrix batches. Classification with SVM and RF was then applied over the eight possible splits of the datasets, exactly as was done in the non-batch adjusted case. We compared the results with the classification accuracies of GRAPE obtained without batch correction (Additional file 1: Figure S6). The post combat classification accuracies were much lower than those obtained by the relative expression methods. All of the pathways had classification accuracies below 0.75 for both SVM and RF (Additional file 1: Figure S6). The results suggest that Combat removed much of the biological variability associated with the tissue types along with the batch effects. We note that although Combat does support inclusion of biological covariates, the software did not allow us to include tissue type in the model because tissue type was confounded with batch membership.

### Identifying differentially expressed pathways between breast cancer subtypes

We investigated the ability of GRAPE to identify pathways that are differentially ordered between three breast cancer subtypes: luminal A, luminal B and basal-like. These subtypes are well established and can be reliably classified using PAM50 classifier [14, 15] as well as other classifiers [16]. For this analysis we used RNA-Seq profiles of 231 luminal A tumors, 127 luminal B tumors and 97 basal-like tumors from TCGA [17]. Additionally, 103 normal breast samples from TCGA were used as the reference samples. For this analysis the set of 397 KEGG and BioCarta pathways having at most 250 genes were considered.

Differential pathway analysis was applied to each pair of subtypes. For each of the tumor samples, pathway scores were calculated for each pathway, as described in the “Methods” section. *P*-values were calculated for each pathway using the two-sided t-test applied to the pathway scores in each subgroup. For the comparison of basal-like and luminal A, 324 out of 397 pathways had Bonferroni adjusted *p*-values below 0.01. All of the pathways that achieved this level of significance were more disordered in the basal-like subtype. Similar results were observed for the comparison of luminal B and luminal A subtypes. In this case 322 pathways achieved the significance threshold and all of these pathways were more disordered in the luminal B subtype. These results suggest that the luminal A subtype is much more similar to normal breast tissue than either of the other two subtypes. For the comparison of basal-like and luminal B, 115 pathways were identified as differentially ordered, with 102 and 13 of these identified as more disordered in the basal-like and luminal B subtypes, respectively. Volcano plots summarizing the three comparisons are shown in Additional file 1: Figure S7.

The results of the GRAPE analysis were compared to those of gene set enrichment analysis (GSEA), which is one of the most popular algorithms for detection of differentially expressed pathways [18]. The recommended threshold of FDR q-value below 0.25 was used to identify differentially expressed pathways. In the comparison of basal-like and luminal A, GSEA identified 26 pathways, with all of them enriched in the basal-like subtype. For the comparison of luminal B with luminal A, 62 pathways were identified with 57 of them enriched in luminal B. Surprisingly, GSEA identified only 1 pathway as differentially expressed between the basal-like and luminal B subtypes. All of the GRAPE and GSEA results are summarized in Table 3. In light of the discrepancies between the GRAPE and GSEA results, a control experiment was performed in which the subtype labels were randomly permuted. Neither GRAPE nor GSEA identified any pathways as statistically significant in the control experiments for any of the three comparisons. To better understand why GRAPE identified so many pathways in each comparison, we calculated the number of differentially expressed genes between each pair of subtypes. Out of 4849 genes occurring at least one pathway, 1597, 496 and 1118 genes were identified at the Bonferroni adjusted 0.01 level in basal-like vs. luminal A, luminal B vs. luminal A and luminal B vs. basal-like, respectively. Additional analyses of the GRAPE and GSEA pathway rankings are presented in the supplemental materials (Additional file 1: Topic S3, Additional file 1: Figure S8 and Additional file 1: Table S6).

**Table 3:** Breast Cancer Pathway Analysis Summary

Met.	Subtypes	# Pathways ^*a*^	Up in class 1	Up in 2	% DE genes
GR	Basal vs. Lum. A	324 (362)	324 (357)	0 (5)	33 (61)
GS	Basal vs. Lum. A	26	26	0	33 (61)
GR	Lum B. vs. Lum. A	322 (369)	322 (369)	0 (0)	10 (32)
GS	Lum B. vs. Lum. A	62	57	5	10 (32)
GR	Basal vs. Lum. B	115 (227)	102 (193)	13 (34)	23 (53)
GS	Basal vs. Lum. B	1	1	0	23 (53)

^a^For GRAPE significance threshold is Bonferroni P below 0.01, threshold of nominal 0.01 *P*-value shown in parentheses. For GSEA significance threshold is FDR q-value below 0.25

### TCGA survival prediction

We evaluated the ability of GRAPE pathway scores to predict patient survival within several cancer subtypes. We compared GRAPE with Gene Set Variation Analysis (GSVA) [9], a method that computes pathway scores for individual samples based on enrichment. GSVA was shown to perform very well at predicting cancer survival in comparison to three other enrichment-based pathway score methods [9], Pathway Level analysis of Gene Expression (PLAGE) [19], single sample GSEA (ssGSEA) [20] and combined z-score [21]. In addition to comparing GRAPE and GSVA, we included the original gene expression (GE) data in the comparison. For this analysis we expanded the pathway set to include all pathways with at most 250 genes from the MSigDB c2 collection (4314 pathways total). This pathway set includes the KEGG and BioCarta pathways as a subset and was chosen in order to more closely match the experimental conditions of the previous comparative analysis in which the full MSigDB c2 collection was used [9].

Survival analysis was performed using ridge regression for two TCGA cancer types: lung adenocarcinomas (LUAD) and breast invasive carcinoma (BRCA). These cancer types were chosen because the TCGA collections include large numbers of both tumor and normal samples. For both cancers, within stage survival analysis was performed for stages two and three. Stage four was omitted due to insufficient samples, and stage one was omitted due to low event frequency. For GRAPE, GSVA and GE, feature selection was performed as described in the methods. Model performance was evaluated by computing the concordance index (CI) of each prediction with the test-set survival data.

**Fig. 3:**
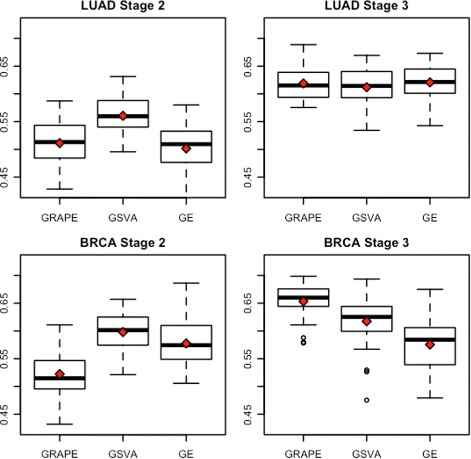
TCGA Survival Prediction. Survival prediction was performed using GRAPE pathway scores, GSVA pathway scores and gene expression (GE). For each feature set, concordance index (CI) of 30 iterations of 3-fold cross validation is shown. Mean CIs are shown in *red*

In the case of LUAD stage 2, GSVA achieved a mean (± sd) CI of 0.56±0.037 (Fig. 3), outperforming both GRAPE (0.51±0.04) and GE (0.50±0.044). GSVA was also the best performing method for BRCA stage 2, achieving a mean CI of 0.60±0.034, compared to 0.58±0.045 for GE and 0.52±0.039 for GRAPE. In the case of BRCA stage 3, GRAPE achieved a mean CI of 0.65±0.032, outperforming GSVA (0.62±0.048) and GE (0.58±0.053). In the case of LUAD stage 3, all of the methods performed similarly, achieving mean CIs of 0.62±0.03 for GRAPE, 0.61±0.04 for GSVA, and 0.62±0.034 for GE. The differential performances between GRAPE and GSVA in 3 out of 4 cases demonstrates that these methods are capturing complementary information about the pathways. Moreover, the fact that GRAPE outperformed both GSVA and GE in one of the cases suggests that GRAPE pathway scores offer a unique representation of gene expression profiles that researchers may consider including in many different prediction contexts.

### Pathway-space visualization of colonic diseases

**Fig. 4:**
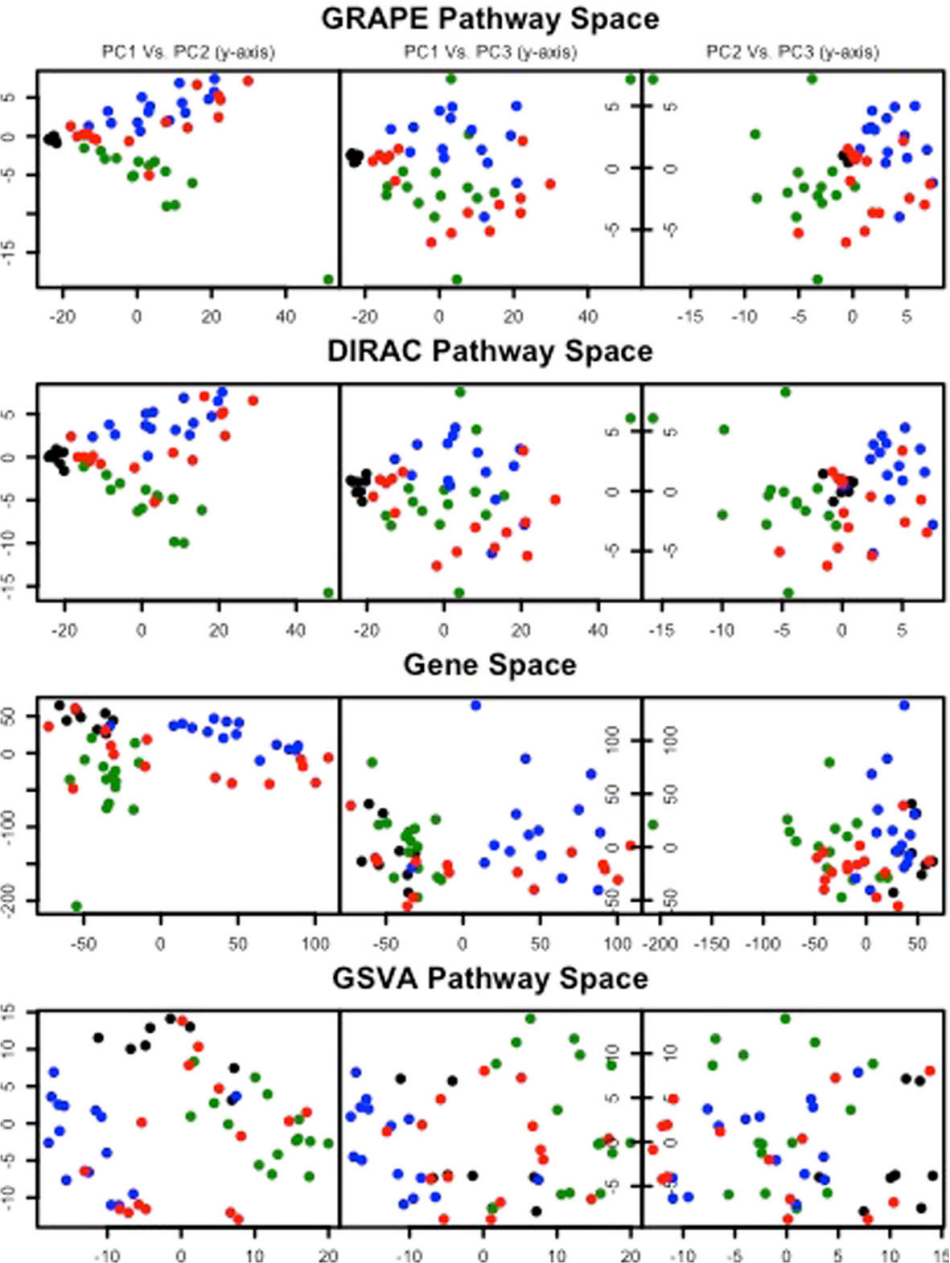
Pairwise plots of first three principal components (PC) for four representations of four colonic tissue types. Normal colon in *black*, IBD samples in *green*, adenomas in *blue*, and colorectal cancer in *red*. For GRAPE, DIRAC and GSVA, pathway scores were calculated for 397 KEGG and BioCarta pathways. The gene space representation consists of the gene expression matrix after removal of genes with standard deviation below 0.01

We evaluated the usefulness of several pathway-space representations on interpretation of three different colon diseases. The dataset, originally presented in [22], consists of 8 normal colon samples, 15 IBD samples, 15 colon adenoma samples and 15 colorectal cancer (CRC) samples. For each of the 397 KEGG and BioCarta pathways that have fewer than 250 genes, three sets of pathway scores were calculated: 1) GRAPE pathway scores using the quadratic weight function (eq. 3), 2) GRAPE pathway scores using a uniform weight function, and 3) GSVA pathway scores. For simplicity the first two pathway space representations are designated by GRAPE and DIRAC, respectively, although this is a slight abuse of notation since the DIRAC method does not contain any notion of pathway scores as originally presented. In addition to these three pathway space representations, a gene space representation consisting of the complete gene expression profiles was also considered. For GRAPE and DIRAC, the normal samples were used as the reference and pathway scores were calculated for all of the samples, including the normal samples themselves. Principal component analysis (PCA) was applied to each matrix of pathway-scores as well as to the gene space. Pairwise plots of the first three principal components (Fig. 4) show that the samples cluster by disease status (black for normal, green for IBD, blue for adenoma, and red for CRC) to varying extents for all four methods. Although none of the four representations display complete separation of the disease types, to our eyes the GRAPE and DIRAC representations both display better separation of subtypes compared to the GSVA and gene space representations.

It is difficult to determine from Fig. 4 alone whether DIRAC or GRAPE achieves better separation of the disease types. To compare the methods further, we removed the IBD samples and repeated the analysis using the normal, adenoma and CRC subtypes. In this case it is evident that the GRAPE pathway scores achieve better separation of the subtypes compared to the DIRAC pathway scores (Additional file 1: Figure S9).

A useful feature of the GRAPE pathway space representation is that the normal samples cluster tightly together and serve as a reference for interpreting the other samples. PC1 appears to capture the magnitude of distance from normal samples and does not discriminate between disease types. PC2 discriminates adenoma samples (above 0) from IBD samples (below 0), with the normal samples tightly clustered around 0. The CRC samples display heterogeneous PC2 scores, with some CRC samples closely resembling normal colon, while most of the others resemble adenomas. PC3 appears to reflect the variability between adenomas and CRC samples, with adenomas mostly having PC3 scores near or above zero and with CRC samples mostly having scores near or below zero.

To see which pathways contributed most to the separation between adenomas and IBD in PC2, we looked at the most extreme loadings (Additional file 1: Table S7, Additional file 1: Figure S10). The largest positive loadings correspond to pathways that have larger pathway scores in adenomas, while the largest negative loadings correspond to pathways that have larger pathways scores in IBD. Eight out of the 20 most negative loadings belonged to pathways involved in immune response and inflammation. Eleven of the 20 most positive loadings were pathways involved in DNA damage and stress response or cell cycle regulation. The most extreme loadings for PC3 are shown in Additional file 1: Table S8. The results suggest that different signaling pathways may be involved in formation of colon adenomas compared to colorectal cancer.

## Discussion

In this article, we first introduced GRAPE as a generalization of DIRAC and then explored the usefulness of GRAPE in several novel applications. First we evaluated the ability of GRAPE to classify four healthy tissues and observed that its performance is almost on par with machine learning methods and superior to DIRAC and PC. Compared with the machine learning methods, GRAPE and DIRAC display vastly superior ability to classify tissue-types across multiple-datasets generated with a common technology platform. Although the performance gap is lower in this setting, GRAPE outperforms DIRAC and PC when three or more tissues are classified. Moreover, we found that the inter-dataset performance of GRAPE remains superior to that of the machine learning methods even after the application of a popular batch correction procedure. None of the methods, however, achieved acceptable classification performance across distinct datasets from different microarray platforms. We showed that pathway scores derived from GRAPE templates of healthy tissues can be used to identify pathways that are differentially expressed between breast cancer subtypes. We performed survival prediction of several cancer subtypes and found that GRAPE pathway-scores outperformed other pathway-space and gene-space representations in some cases. Finally we showed that GRAPE pathway-space representations of samples enable good characterization of the separation between three colonic diseases compared to other pathway-space representations.

### Classification of healthy tissues using individual pathways

It is interesting that the majority of KEGG and BioCarta pathways were able to discriminate among four healthy tissue-types using multiple classification techniques. This observation suggests that different biological phenotypes are distinguished by numerous, sometimes subtle, changes in gene expression, rather than by pronounced differences in the expression of a few genes. Although this may not be surprising, it has important implications for choosing a method for pathway level analysis. Specifically, popular methods based on enrichment analysis that emphasize the most differentially expressed genes may be less powerful for some applications that call for sensitive detection of meaningful biological differences. By contrast, template-generating methods, including but not limited to those presented here, characterize the typical profile of all pathways an investigator may be interested in, and allow for detection of all differentially expressed pathways regardless of whether the genes in the pathway are among the most differentially expressed across the genome.

The observation that GRAPE performs nearly as well as RF and SVM demonstrates the rich information content contained in the relative rankings of gene expression values. Of course we do not mean to suggest that the information content is identical to that contained in absolute gene expression. Indeed, it is easy to imagine a situation in which the relative rankings of a set of genes are identical but yet there are profound differences in the absolute levels of expression. Rather, our analysis suggests that the information content within the relative rankings is sufficient for many applications. Previous analyses have shown that relative expression analyses perform well for genome-wide classification [12]. Here we extend this observation to classification using pre-specified subsets of genes that comprise established biological networks.

### Single platform multi-dataset classification of healthy tissues

In the single platform multi-dataset analyses (Fig. 2, Additional file 1: Figure S3), PC achieved the best classification performance for 20% (Affymetrix) and 25% (Agilent) of the pathways. This is in stark contrast to the single-dataset analysis in which PC was not the best performer for any pathway. Although the average classification performance of PC was lower in the multi-dataset analyses, the decrease was smaller than for the other four methods. One explanation for this disparity is that PC performs better on microarray data compared to RNA-Seq data, as the former is typically normalized before standardization while the latter is presented in units of RPKM (Reads per Kilobase of Transcript per Million mapped Reads) and spans a wide range values, likely leading to excess kurtosis. This suggests that the performance of PC on RNA-Seq data may be improved by implementing quantile normalization instead of the minimalistic standardization procedure used here.

The better results from PC indicate that the superior performance of GRAPE and DIRAC relative to RF and SVM cannot be wholly attributed to use of gene expression rankings as opposed to absolute expression measurements. We speculate that the improved performance of all three methods compared to the machine learning methods stems from the fact that the machine learning pathways determine variable importance from the train set exclusively and are therefore unable to account that the discriminating value (i.e., importance) of the genes may change considerable in the presence of systematic batch effects. By contrast the template-based classification methods do not determine variable importance within the train set. These methods are therefore able to consider all of the genes equally, and can better utilize genes that have the greatest discriminating value in the presence of batch effects, even if these are not the same as the most discriminatory genes within the training set alone. Consistent with this is our observation that small percentage of genes account for large percentage of inconsistent gene-pairs, suggesting that a minority of genes display severe inter-dataset inconsistencies. It is possible that RF and SVM are more vulnerable to these inconsistencies because they may assign importance to only a small subset of genes.

The idea that it may be advantageous to use the maximum number of genes when classifying tissue types across multiple datasets also explains the comparative improvement of DIRAC relative to GRAPE in the multi-dataset analyses. Whereas in the single dataset analysis of four classes GRAPE outperformed DIRAC (with ties not counted) in 387 out of 395 pathways, in the Affymetrix analysis of three classes GRAPE outperformed DIRAC in 221/381 pathways and in 141/260 pathways in the Agilent analysis of two classes. Perhaps the inter-dataset performance of GRAPE is diminished relative to DIRAC because the weight function reduces the impact of some of the genes. The number of classes in each analysis is likely to have affected the performance differences between DIRAC and GRAPE. The results support the trend that the relative performance of GRAPE compared to DIRAC improves as the number of classes increases. Single-dataset analyses with different numbers of classes further support this trend (Additional file 1: Figure S11).

### Between platform multi-dataset classification of healthy tissues

The accuracy of inter-platform multi-dataset analyses was very poor for all methods. Even though SVM and PC comparatively outperformed GRAPE and DIRAC in this setting, we do not consider this to be important because all five methods had average classification accuracies below 50% across all pathways, and none achieved classification performance of 90% for any pathway. The results indicate that between-platform effects are considerably more severe than batch effects between datasets using the same platform.

According to the 2006 Microarray Analysis of Quality Control studies [4, 5], the causes of inter-platform effects likely include differences in probe-sequence and unreliable quantification of poorly expressed genes. If genome-wide gene-expression profiling using multiple platforms is to become routine in clinical settings, the reliability of the data must be improved considerably. One possible approach is to subset the genome so that it only includes gene-pairs that are ranked consistently across inter-platform datasets for similar biological samples. The feasibility of this approach would first need to be evaluated by determining whether such a subset exists across many different datasets of the same healthy tissue types. This suggestion is consistent with the theme behind using gene-rankings instead of absolute expression: it is better to consider a smaller quantity of information that is reliable than a larger quantity that is not. Perhaps, if combined with streamlining the technical procedure as much as possible to mitigate “pre-analytical” variability, this sub-setting strategy could enable reliable identification of abnormal pathways using GRAPE or other template-generating methods.

### Inter-dataset classification with Combat Vs. GRAPE

The poor inter-dataset classification performances of RF and SVM after application of Combat highlights the difficulty of distinguishing biological variability from experimental variability when the two are confounded. Presumably the classification performances would have been much better in a situation in which each of the datasets (“batches”) contained samples of each tissue type. Our results suggest that rank-based methods may offer a strong alternative for robust classification in same-platform situations where the biological effects are not balanced across batches. Examples of such situations would be meta-analyses that seek to improve statistical power by pooling many smaller studies.

### GRAPE vs. GSEA

The discrepancy in the number of differentially expressed pathways between pairs of breast cancer subtypes identified by GRAPE compared to GSEA highlights the differences between template-based pathway methods and enrichment methods. In GRAPE the identification of a pathway as differentially expressed depends only on the expression levels of the genes within the pathways. By contrast, in GSEA, a pathway is identified as differentially expressed if the genes within the pathway are over-represented near the top of a list ranking all of the genes in the genome according to the degree of differential expression. This implies that the identification of a pathway as differentially expressed or not depends in part on the gene expression values of genes that are not in the pathway. Another way to express this difference is that enrichment methods are competitive in the sense that each pathway is implicitly compared to other pathways as part of the enrichment determination, whereas template-based methods are completely non-competitive. Because of this difference the two methods offer complementary information and may each be preferred in certain situations. For example, GRAPE may offer more sensitive detection of differentially expressed pathways whose genes may not be among the most differentially expressed across the genome. On the other hand, GSEA may be preferred in situations where researchers seek to identify only the most likely drivers of a disease rather than all differentially expressed pathways.

### Pathway scores enable pathway-space portrayal of samples relative to a reference

One of the limitations of any classification algorithm is that such algorithms require predetermined class definitions. In the case of a complex disease such as cancer, however, there is significant heterogeneity within many of the established subtypes and uncovering the structure in this heterogeneity can be critical to predicting drug response or prognosis. A potentially powerful application of GRAPE is to identify biological patterns at the pathway level by comparing the samples of interest to a suitable reference template. This is accomplished by calculating pathway scores for individual samples that quantify the dissimilarity of each pathway within the sample from the behavior observed within the reference collection. By considering both the reference template and the amount of variability of the reference samples around the template for each pathway, abnormally behaving pathways in an individual patient can be identified. Mathematically, this can be thought of as a transformation from the space of gene expression values, to a reduced dimensional space of pathway scores. The pathway scores were designed to be analogous to one-sided z-scores so that abnormal pathways can be detected by surveying the pathway scores.

This pathway-space representation enables consideration of the interactions of different pathways. For example, a particular cancer subtype may be defined by the abnormal behavior of two pathways and lack of abnormal behavior of a third pathway. Although the pathway scores are derived from the gene-expression values, they contain information about the interactions of genes that may not be accessible to prediction algorithms based on individual genes. As such the pathway scores can be used as an additional set of features for supervised prediction or unsupervised clustering. We demonstrate the usefulness and novelty of the GRAPE pathway space representation at predicting patient survival within several cancer subtypes. Our results suggest that in some contexts GRAPE pathways scores capture useful information from the normal samples that is not present in other representations. Our analysis of colonic diseases relative to healthy colon tissue further demonstrates the use of pathway scores to cluster different pathological states and identify pathways that are differentially perturbed among the subtypes. Additional potential applications include drug sensitivity analysis applied to pharmacogenomics datasets and unsupervised clustering to identify new cancer subtypes.

## Conclusion

We presented a new class of pathway methods that use healthy reference samples to quantify the abnormality of individual pathological samples. We observed that methods based on pairwise rankings offer superior signal to noise ratios in the presence of single platform inter-dataset batch effects compared to methods based on absolute gene expression. Our classification results suggest that GRAPE is the method best-suited for identification of abnormal pathway behavior, as it is the only template-based method that can discriminate among different tissue types with accuracies similar to state of the art machine learning techniques within a single dataset. GRAPE offers a convenient, reliable, and versatile procedure for determining whether a network of genes is behaving abnormally. GRAPE pathway scores provide researchers with a unique perspective of patient transcription profiles that may lead to improvements in the prediction performances of a wide range of personalized medicine applications.

## Additional file

Additional file 1 This file contains all supplementary materials, including supplementary topics S1-S3, supplementary tables S1-S8 and supplementary figures S1-S11. (DOCX 1044 kb)

## Abbreviations

ARDE-(S/N): Absolute rate of differential expression (standardized/non-standardized)
BRCA: Breast invasive carcinoma
CRC: Colorectal cancer
DIRAC: Differential rank conservation
DOM: Differential ordering measure
GRAPE: Gene-ranking analysis of pathway expression
GSEA: Gene set enrichment analysis
GSVA: Gene set variation analysis
GTEx: The genotype-tissue expression project
IBD: Inflammatory bowel disease
LUAD: Lung adenocarcinoma
PC: Pathway centroid
PLAGE: Pathway level analysis of gene expression
RF: Random forest
RRDE: Relative rate of differential expression
ssGSEA: Single sample GSEA
SVM: Support vector machine
TCGA: The cancer genome atlas
