# Supplementary material for: GRAPE: a pathway template method to characterize tissue-specific functionality from gene expression profiles
Source: BMC Bioinformatics. 2017 Jun 26;18:317. doi: 10.1186/s12859-017-1711-z (PMC5485588; doi:10.1186/s12859-017-1711-z)
Supplement: Supplementary file 1 — This file contains all supplementary materials, including supplementary topics S1-S3, supplementary tables S1-S8 and supplementary figures S1-S11. (DOCX 1044 kb) [file 12859_2017_1711_MOESM1_ESM.docx]

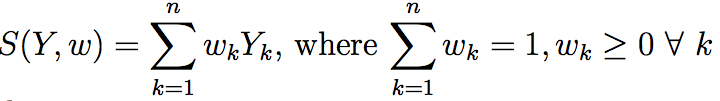

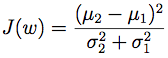

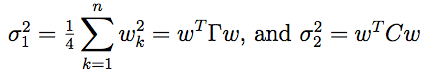

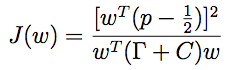

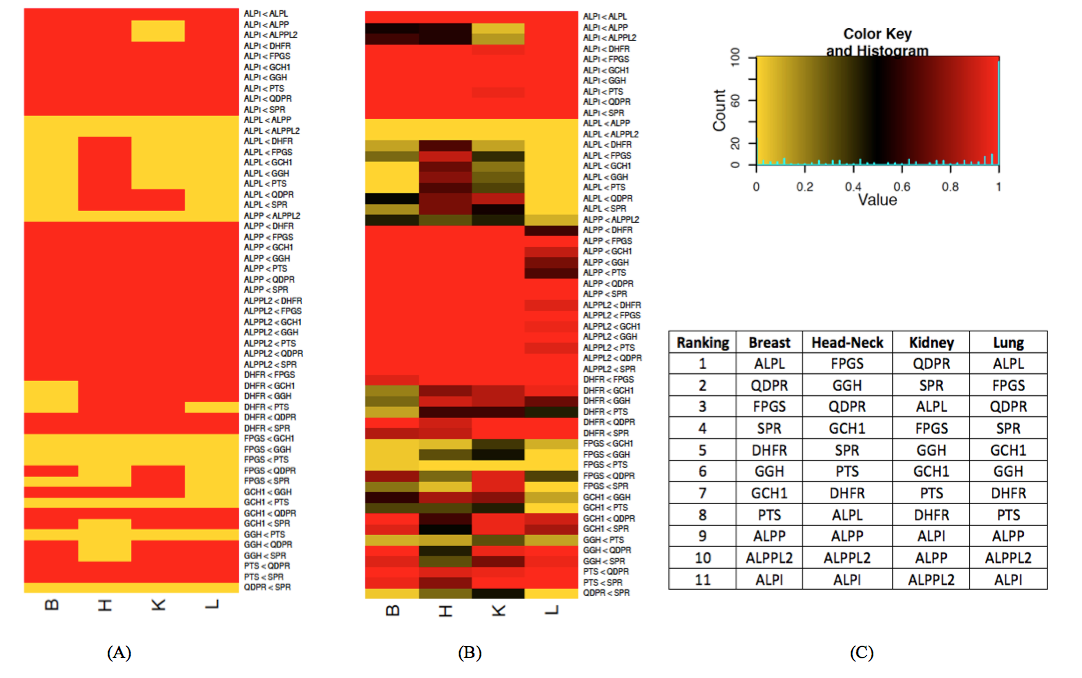

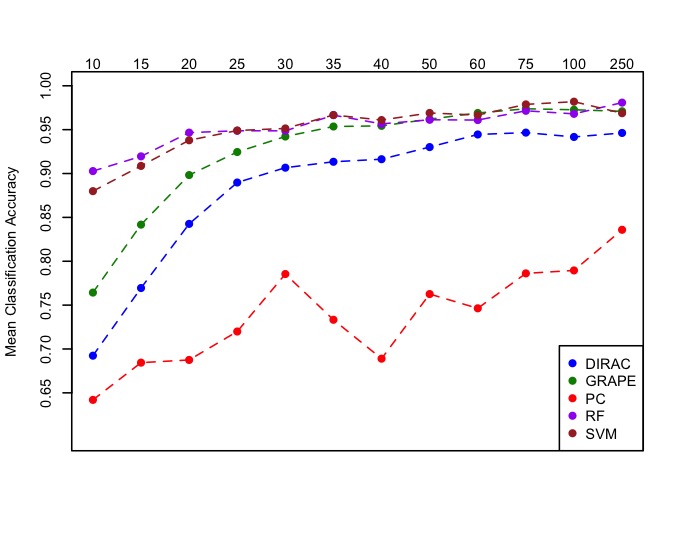

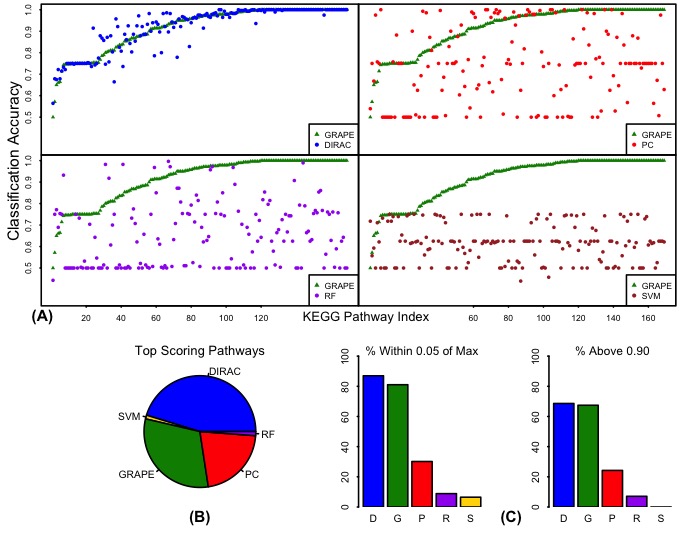

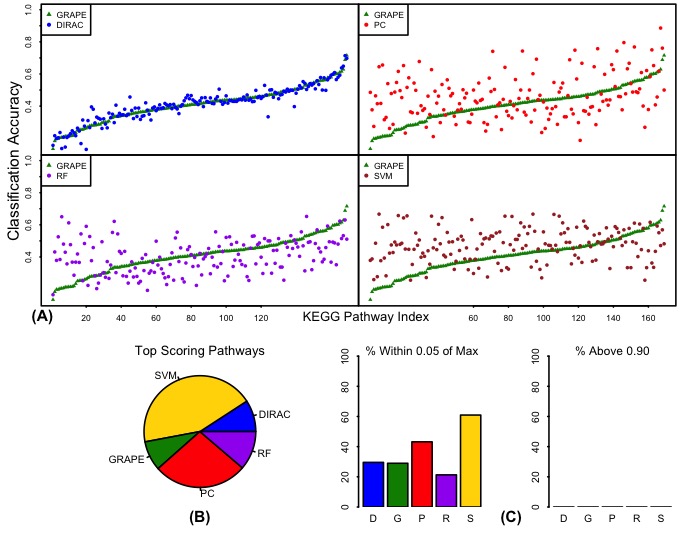

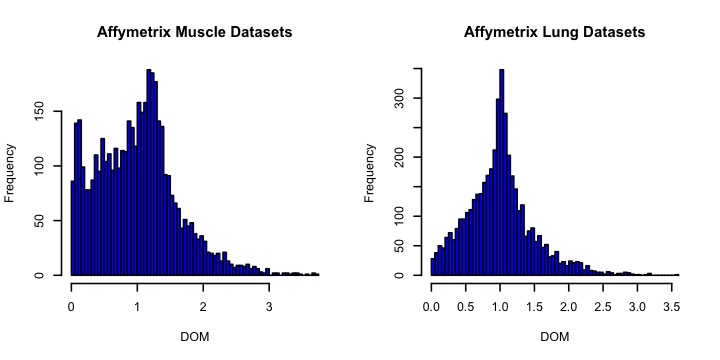

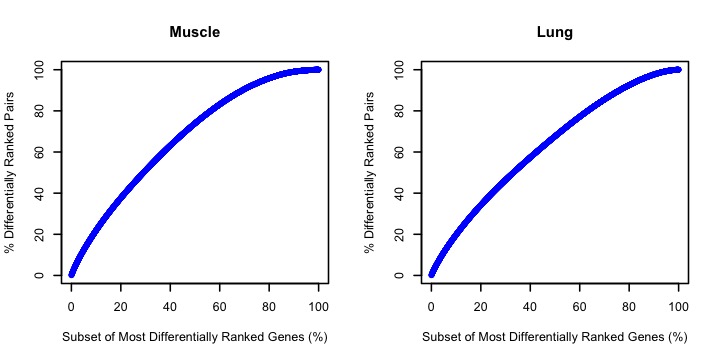

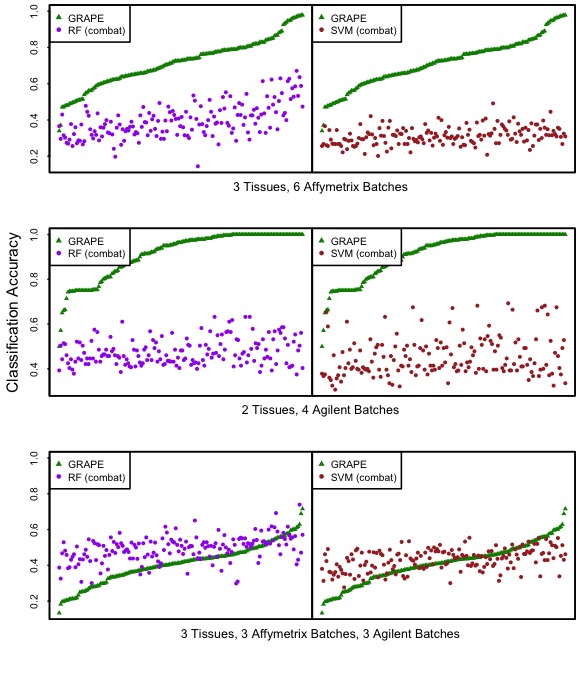

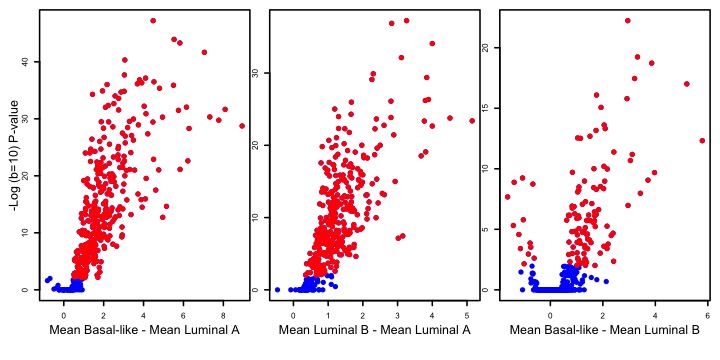

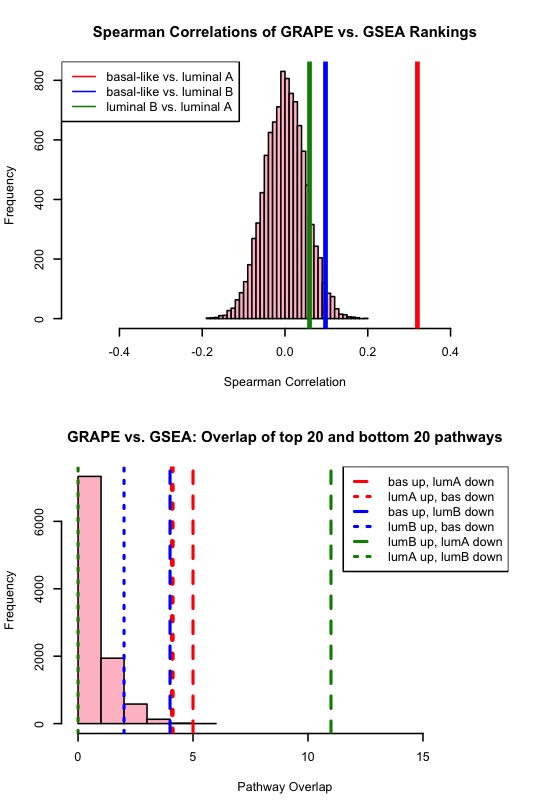

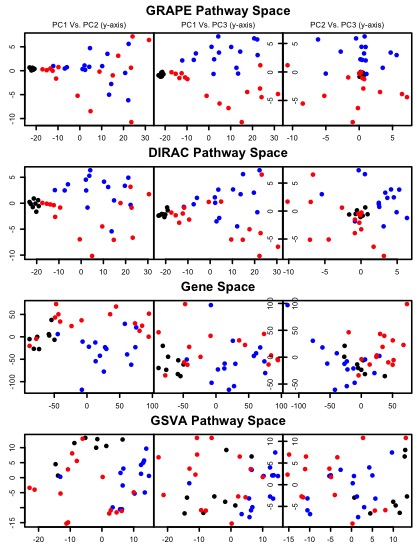

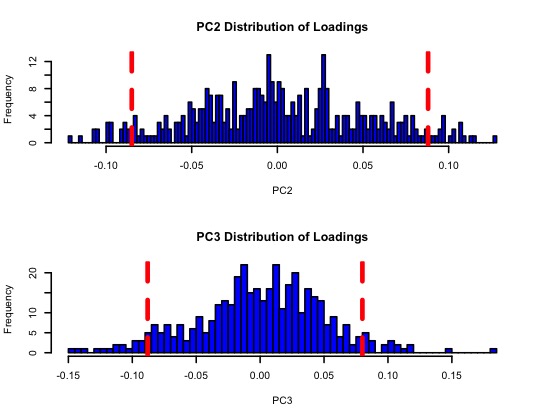
**Supplementary Materials for GRAPE**

**Topic S1: Optimization of weight function**

Let *t* and *p* be the binary template and probability template for a particular tissue type *T*. Without loss of generality, we orient all pairs so that *t_k_ =* 1, for *k = 1,2, …, n*. Let *Y* be the binary-valued vector representation of a sample. We define the matching score of *Y* to *t* as:


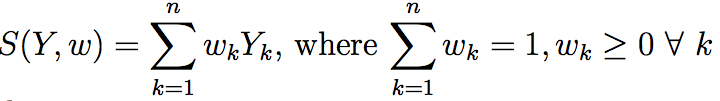


We consider two that the sample belongs to one of two possible distributions.

D_1_: The null distribution. In this case we assume that for all *k, Y_k_* are i.i.d. Bernoulli(1/2) r.v.

D_2_: Tissue type *T* distribution. In this case, *Y_k_* ~ Bernoulli(*p_k_*) and the covariance of (*Y_i_, Y_j_*) is given by the covariance matrix **C**. The sample covariances of the reference samples are used to estimate **C**.

We seek to find *w* that maximizes the distances between D_1_ and D_2_. We write


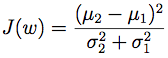


where μ_1_, μ_2_ and σ_1_, σ_2_ are the mean and standard deviation for each distribution.

Solving for these values, we have *μ_1_ = ½, μ_2_ = w•p*. Defining **Γ** to be the *n x n* matrix with ¼ on the diagonal and zero elsewhere, we have:


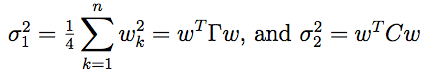


We can re-write the objective function as


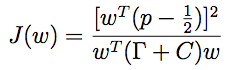


Borrowing from Markowitz Portfolio Theory in finance [[1](#_ENREF_1)], we can use quadratic programming to solve for *w* that maximizes *q*(p-1/2)^T^w – w^T^(****Γ****+****C****)w*, for a given risk tolerance parameter *q*. By varying *q* over a range we can generate a curve representing the efficient mean-variance frontier (EMVF). Next we argue that the solution *w_opt_*that maximizes *J(w)* must occur along the EMVF. To see this, consider that if *w_opt_*does not occur along the frontier, then there exists w* along EMVF for which *(p-1/2)^T^w* = (p-1/2)^T^w_opt_* and *w*^T^(****Γ****+****C****)w* < w_opt_^T^(****Γ****+****C****)w_opt_ .* It follows that *J(w*) > J(w_opt_),* which contradicts the optimality of *w_opt_*. So to solve for *w_opt_*, we generated the EMVF and then performed a line search to find the *w* that maximized *J*. For each optimization the weights were also constrained so that no single gene-pair is weighted more than *1.5/n*. We found that this constraint led to improved classification performances.

We compared the classification performance of healthy tissues using the optimized weight function versus a few intuitively chosen weight functions. In addition to the quadratic weight function, *(x-0.5)^2^,* we considered a quartic weight function*, (x-0.5)^4^,* a linear weight function, *|x-0.5|*, and also to a step function that is zero between 0.4 and 0.6 and 1 elsewhere. We considered the set of KEGG pathways having between 10 and 35 genes. We observed that the classification accuracies using the various weight functions were nearly indistinguishable from those of the optimal weight function. The lone exception was the step function, which achieved slightly lower accuracies than the other weight functions. We further evaluated the objective function scores of the various weight functions for 6 randomly selected KEGG pathways of various sizes (evenly spaced between 15 and 40 genes). In all of these pathways the quadratic weight function achieved the second highest objective function score, second only to the optimized weight function. The quadratic weight function score was within 2% of the optimal weight function score in 3 out 6 pathways. This suggests that the objective function landscape for this optimization problem is very flat at the top, permitting many good solutions. Considering that the optimal weight function achieved negligibly better classification performance at a dramatic increase in computational cost, we chose to proceed with the quadratic weight function as the default weight function for GRAPE.

**Topic S2: TCGA Outliers**

Breast normal sample number 88 and head-neck normal sample number 24 were discarded from analysis due to suspicion of being outliers. The unusual behavior of these two samples was observed within tissue-specific heatmaps of pathway scores. Breast sample 88 and head-neck sample 24 displayed large pathways scores for nearly all of the pathways considered. No other samples displayed this pathway-score profile.

**Topic S3: Analysis of GRAPE and GSEA pathway rankings for BRCA subtype comparisons**

For each comparison we ranked the pathways from the most significantly upregulated in class 1 (rank = 1) to the most significantly upregulated in class 2 (rank = 397). We evaluated the similarity between the GRAPE and GSEA rankings using two metrics. First, we calculated the spearman correlation between the GRAPE and GSEA rankings. Second, we counted the number of overlapping pathways within the top 20 and bottom 20 pathways of each list. To assess the significance of the results, null distributions for both metrics were generated from 10,000 pairs of randomly ranked lists. The results are shown in Figure S7. The strongest correlation was observed for the basal-like vs. luminal A comparison, for which the spearman correlation was 0.32. Spearman correlations of 0.098 and 0.059 were observed for the basal-like vs. luminal B comparison and the luminal B vs. luminal A comparison, respectively. Interestingly, the strongest overlap was observed for pathways upregulated in luminal B relative to luminal A, as 11 out of the top 20 pathways were shared between GSEA and GRAPE. For the same comparison in the reverse direction, i.e., pathways upregulated in luminal A relative to luminal B, zero pathways overlapped between GSEA and GRAPE. The lack of overlap in the luminal A up direction may explain why the overall correlation was weak for this comparison, despite the very strong overlap at the top of the luminal B up direction. For the basal-like vs. luminal A comparison, 5 pathways overlapped in the basal-like up direction and 4 pathways overlapped in the luminal A up direction. These overlaps are significant compared to the null distribution, for which 98.6% of the random lists had <= 3 overlapping pathways. For the basal-like vs. luminal B comparison, 4 pathways overlapped in the basal-like up direction and 2 pathways overlapped in the luminal B up direction. The overlapping pathways from each comparison are highlighted in Table S5.

**Supplemental Tables and Figures**

**Table S1: Classification Results of Full Pathway Set**

**(All KEGG and BioCarta pathways with <= 250 genes)**

|  | Method | % top performer | % within 0.05 of top performer | % above 0.90 |
| --- | --- | --- | --- | --- |
| TCGA four tissues, one dataset | GRAPE | 14.8 | 72.8 | 71.8 |
|  | DIRAC | 0.3 | 41.8 | 52.9 |
|  | PC | 0.0 | 1.5 | 7.8 |
|  | RF | 42.4 | 99.0 | 91.4 |
|  | SVM | 42.5 | 95.2 | 89.9 |
| Affymetrix three tissues, 6 datasets | GRAPE | 41.2 | 74.1 | 5.8 |
|  | DIRAC | 29.6 | 68.5 | 5.3 |
|  | PC | 17.6 | 27.2 | 3.8 |
|  | RF | 3.7 | 12.1 | 0.0 |
|  | SVM | 7.9 | 13.1 | 0.25 |
| Agilent two tissues, 4 datasets | GRAPE | 33.5 | 77.3 | 57.9 |
|  | DIRAC | 36.3 | 74.8 | 58.9 |
|  | PC | 24.3 | 35.5 | 30.7 |
|  | RF | 4.0 | 12.6 | 11.1 |
|  | SVM | 1.8 | 5.8 | 0.5 |
| 3 tissues, 3 Affymetrix datasets, 3 Agilent datasets | GRAPE | 7.9 | 25.9 | 0 |
|  | DIRAC | 9.2 | 24.9 | 0 |
|  | PC | 26.2 | 42.6 | 0 |
|  | RF | 13.1 | 24.7 | 0 |
|  | SVM | 43.6 | 58.9 | 0 |

**Table S2: ARDE and RRDE of Affymetrix Datasets**

|  | **ARDE-N** | **ARDE-S** | **RRDE** |
| --- | --- | --- | --- |
| L1_L2 | 0.99 | 0.92 | 0.27 |
| M1_M2 | 0.92 | 0.67 | 0.2 |
| C1_C2 | 0.99 | 0.74 | 0.32 |
| L1_M1 | 0.78 | 0.78 | 0.33 |
| L1_M2 | 0.81 | 0.82 | 0.34 |
| L1_C1 | 0.9 | 0.76 | 0.24 |
| L1_C2 | 0.99 | 0.78 | 0.34 |
| L2_M1 | 0.98 | 0.93 | 0.41 |
| L2_M2 | 0.99 | 0.95 | 0.41 |
| L2_C1 | 0.99 | 0.9 | 0.35 |
| L2_C2 | 0.96 | 0.81 | 0.44 |
| M1_C1 | 0.96 | 0.81 | 0.31 |
| M1_C2 | 0.99 | 0.79 | 0.4 |
| M2_C1 | 0.83 | 0.84 | 0.33 |
| M2_C2 | 1 | 0.79 | 0.48 |
| **Mean Homo-tissue** | **0.97** | **0.78** | **0.26** |
| **Mean Hetero-tissue** | **0.93** | **0.83** | **0.37** |
| **Ratio of means** | **0.96** | **1.07** | **1.39** |

**Table S3: ARDE and RRDE of Agilent Datasets**

|  | **ARDE-N** | **ARDE-S** | **RRDE** |
| --- | --- | --- | --- |
| L1_L2 | 0.72 | 0.76 | 0.19 |
| M1_M2 | 0.99 | 0.92 | 0.25 |
| L1_M1 | 0.97 | 0.9 | 0.39 |
| L1_M2 | 0.95 | 0.92 | 0.33 |
| L2_M1 | 0.98 | 0.9 | 0.39 |
| L2_M2 | 0.94 | 0.94 | 0.33 |
| **Mean Homo-tissue** | **0.86** | **0.84** | **0.22** |
| **Mean Hetero-tissue** | **0.96** | **0.92** | **0.36** |
| **Ratio of means** | **1.12** | **1.09** | **1.63** |

**Table S4: ARDE and RRDE of Mixed Affymetrix/Agilent Datasets**

|  | **ARDE-N** | **ARDE-S** | **RRDE** |
| --- | --- | --- | --- |
| L1_L2 | 0.96 | 0.93 | 0.44 |
| M1_M2 | 0.89 | 0.96 | 0.28 |
| C1_C2 | 0.99 | 0.5 | 0.71 |
| L1_M1 | 1 | 0.94 | 0.38 |
| L1_M2 | 0.94 | 0.94 | 0.42 |
| L1_C1 | 0.99 | 0.92 | 0.34 |
| L1_C2 | 1 | 0.72 | 0.87 |
| L2_M1 | 0.99 | 0.94 | 0.43 |
| L2_M2 | 0.95 | 0.95 | 0.32 |
| L2_C1 | 0.99 | 0.91 | 0.38 |
| L2_C2 | 1 | 0.74 | 0.88 |
| M1_C1 | 0.85 | 0.83 | 0.33 |
| M1_C2 | 1 | 0.52 | 0.87 |
| M2_C1 | 0.91 | 0.88 | 0.38 |
| M2_C2 | 0.99 | 0.43 | 0.89 |
| **Mean Homo-tissue** | **0.95** | **0.8** | **0.48** |
| **Mean Hetero-tissue** | **0.97** | **0.81** | **0.54** |
| **Ratio of means** | **1.02** | **1.01** | **1.14** |

**Table S5: Top Pathways from BRCA Subtype Comparisons**

Overlapping pathways are highlighted in light blue. Note, in the case of luminal A up, basal-like down, GRAPE only identified 14 pathways, and in the case of luminal A up, luminal B down, GRAPE only identified 2 pathways. The remaining 6 and 18 pathways in these cases, respectively, are actually the least-significant pathways in the reverse direction. These pathways are presented in light gray text to emphasize that they do not belong in their respective categories, but are included only for completeness of the analysis.

| **Basal-like Up, Luminal A Down** | |  |
| --- | --- | --- |
| **Rank** | **GSEA** | |
| 1 | KEGG_GLYCOSPHINGOLIPID_BIOSYNTHESIS_LACTO_AND_NEOLACTO_SERIES | |
| 2 | BIOCARTA_ACTINY_PATHWAY | |
| 3 | KEGG_DNA_REPLICATION | |
| 4 | KEGG_CYSTEINE_AND_METHIONINE_METABOLISM | |
| 5 | KEGG_CELL_CYCLE | |
| 6 | BIOCARTA_G2_PATHWAY | |
| 7 | KEGG_HOMOLOGOUS_RECOMBINATION | |
| 8 | KEGG_P53_SIGNALING_PATHWAY | |
| 9 | KEGG_ONE_CARBON_POOL_BY_FOLATE | |
| 10 | BIOCARTA_RB_PATHWAY | |
| 11 | BIOCARTA_RANMS_PATHWAY | |
| 12 | BIOCARTA_DNAFRAGMENT_PATHWAY | |
| 13 | KEGG_GLYOXYLATE_AND_DICARBOXYLATE_METABOLISM | |
| 14 | KEGG_PYRIMIDINE_METABOLISM | |
| 15 | KEGG_GALACTOSE_METABOLISM | |
| 16 | BIOCARTA_PTC1_PATHWAY | |
| 17 | BIOCARTA_MCM_PATHWAY | |
| 18 | KEGG_SPLICEOSOME | |
| 19 | KEGG_RNA_DEGRADATION | |
| 20 | BIOCARTA_CELLCYCLE_PATHWAY | |
| **Luminal A Up, Basal-like Down** | |  |
| **Rank** | **GSEA** | |
| 1 | KEGG_PEROXISOME | |
| 2 | BIOCARTA_HER2_PATHWAY | |
| 3 | KEGG_VASOPRESSIN_REGULATED_WATER_REABSORPTION | |
| 4 | BIOCARTA_P35ALZHEIMERS_PATHWAY | |
| 5 | KEGG_TYPE_II_DIABETES_MELLITUS | |
| 6 | BIOCARTA_WNT_PATHWAY | |
| 7 | KEGG_SPHINGOLIPID_METABOLISM | |
| 8 | BIOCARTA_EGFR_SMRTE_PATHWAY | |
| 9 | BIOCARTA_LEPTIN_PATHWAY | |
| 10 | KEGG_CIRCADIAN_RHYTHM_MAMMAL | |
| 11 | KEGG_ENDOCYTOSIS | |
| 12 | KEGG_ABC_TRANSPORTERS | |
| 13 | BIOCARTA_MTOR_PATHWAY | |
| 14 | BIOCARTA_BAD_PATHWAY | |
| 15 | BIOCARTA_RARRXR_PATHWAY | |
| 16 | BIOCARTA_TGFB_PATHWAY | |
| 17 | BIOCARTA_P38MAPK_PATHWAY | |
| 18 | BIOCARTA_HCMV_PATHWAY | |
| 19 | BIOCARTA_CFTR_PATHWAY | |
| 20 | BIOCARTA_EXTRINSIC_PATHWAY | |
| **Basal-like Up, Luminal B Down** | |  |
| **Rank** | **GSEA** | |
| 1 | KEGG_GLYCOSPHINGOLIPID_BIOSYNTHESIS_LACTO_AND_NEOLACTO_SERIES | |
| 2 | KEGG_PATHOGENIC_ESCHERICHIA_COLI_INFECTION | |
| 3 | KEGG_DORSO_VENTRAL_AXIS_FORMATION | |
| 4 | KEGG_P53_SIGNALING_PATHWAY | |
| 5 | KEGG_CYSTEINE_AND_METHIONINE_METABOLISM | |
| 6 | BIOCARTA_ACTINY_PATHWAY | |
| 7 | KEGG_NON_SMALL_CELL_LUNG_CANCER | |
| 8 | BIOCARTA_PARKIN_PATHWAY | |
| 9 | KEGG_AXON_GUIDANCE | |
| 10 | KEGG_RENAL_CELL_CARCINOMA | |
| 11 | BIOCARTA_SARS_PATHWAY | |
| 12 | KEGG_ERBB_SIGNALING_PATHWAY | |
| 13 | KEGG_WNT_SIGNALING_PATHWAY | |
| 14 | KEGG_GLIOMA | |
| 15 | BIOCARTA_CYTOKINE_PATHWAY | |
| 16 | KEGG_CELL_CYCLE | |
| 17 | KEGG_NOTCH_SIGNALING_PATHWAY | |
| 18 | KEGG_ONE_CARBON_POOL_BY_FOLATE | |
| 19 | BIOCARTA_ETS_PATHWAY | |
| 20 | KEGG_MELANOGENESIS | |
| **Luminal B Up, Basal-like Down** | |  |
| **Rank** | **GSEA** | |
| 1 | KEGG_VASOPRESSIN_REGULATED_WATER_REABSORPTION | |
| 2 | KEGG_PEROXISOME | |
| 3 | BIOCARTA_HER2_PATHWAY | |
| 4 | KEGG_ENDOCYTOSIS | |
| 5 | BIOCARTA_RARRXR_PATHWAY | |
| 6 | KEGG_GLYCOSYLPHOSPHATIDYLINOSITOL_GPI_ANCHOR_BIOSYNTHESIS | |
| 7 | BIOCARTA_MTOR_PATHWAY | |
| 8 | BIOCARTA_IGF1MTOR_PATHWAY | |
| 9 | BIOCARTA_EGFR_SMRTE_PATHWAY | |
| 10 | BIOCARTA_AKAPCENTROSOME_PATHWAY | |
| 11 | BIOCARTA_CFTR_PATHWAY | |
| 12 | BIOCARTA_WNT_PATHWAY | |
| 13 | KEGG_SPHINGOLIPID_METABOLISM | |
| 14 | BIOCARTA_LEPTIN_PATHWAY | |
| 15 | BIOCARTA_P35ALZHEIMERS_PATHWAY | |
| 16 | KEGG_BIOSYNTHESIS_OF_UNSATURATED_FATTY_ACIDS | |
| 17 | KEGG_ALDOSTERONE_REGULATED_SODIUM_REABSORPTION | |
| 18 | BIOCARTA_EIF4_PATHWAY | |
| 19 | KEGG_PANTOTHENATE_AND_COA_BIOSYNTHESIS | |
| 20 | KEGG_TYPE_II_DIABETES_MELLITUS | |
| **Luminal B Up, Luminal A Down** | |  |
| **Rank** | **GSEA** | |
| 1 | KEGG_CELL_CYCLE | |
| 2 | KEGG_OOCYTE_MEIOSIS | |
| 3 | KEGG_DNA_REPLICATION | |
| 4 | BIOCARTA_ATRBRCA_PATHWAY | |
| 5 | BIOCARTA_G2_PATHWAY | |
| 6 | KEGG_HOMOLOGOUS_RECOMBINATION | |
| 7 | KEGG_MISMATCH_REPAIR | |
| 8 | KEGG_NUCLEOTIDE_EXCISION_REPAIR | |
| 9 | BIOCARTA_CELLCYCLE_PATHWAY | |
| 10 | KEGG_ONE_CARBON_POOL_BY_FOLATE | |
| 11 | KEGG_PYRIMIDINE_METABOLISM | |
| 12 | BIOCARTA_PTC1_PATHWAY | |
| 13 | KEGG_BASE_EXCISION_REPAIR | |
| 14 | BIOCARTA_MCM_PATHWAY | |
| 15 | BIOCARTA_RANMS_PATHWAY | |
| 16 | BIOCARTA_AKAP95_PATHWAY | |
| 17 | BIOCARTA_SKP2E2F_PATHWAY | |
| 18 | BIOCARTA_P27_PATHWAY | |
| 19 | KEGG_PROGESTERONE_MEDIATED_OOCYTE_MATURATION | |
| 20 | BIOCARTA_PROTEASOME_PATHWAY | |
| **Luminal A Up, Luminal B Down** | |  |
| **Rank** | **GSEA** | |
| 1 | KEGG_VASCULAR_SMOOTH_MUSCLE_CONTRACTION | |
| 2 | BIOCARTA_EGF_PATHWAY | |
| 3 | KEGG_FOCAL_ADHESION | |
| 4 | BIOCARTA_ALK_PATHWAY | |
| 5 | BIOCARTA_PDGF_PATHWAY | |
| 6 | KEGG_ETHER_LIPID_METABOLISM | |
| 7 | KEGG_GLYCOSAMINOGLYCAN_BIOSYNTHESIS_CHONDROITIN_SULFATE | |
| 8 | BIOCARTA_EDG1_PATHWAY | |
| 9 | BIOCARTA_PPARA_PATHWAY | |
| 10 | KEGG_ARRHYTHMOGENIC_RIGHT_VENTRICULAR_CARDIOMYOPATHY_ARVC | |
| 11 | BIOCARTA_RAC1_PATHWAY | |
| 12 | KEGG_ECM_RECEPTOR_INTERACTION | |
| 13 | BIOCARTA_MYOSIN_PATHWAY | |
| 14 | KEGG_COMPLEMENT_AND_COAGULATION_CASCADES | |
| 15 | KEGG_TGF_BETA_SIGNALING_PATHWAY | |
| 16 | BIOCARTA_CARDIACEGF_PATHWAY | |
| 17 | KEGG_CALCIUM_SIGNALING_PATHWAY | |
| 18 | KEGG_DILATED_CARDIOMYOPATHY | |
| 19 | BIOCARTA_NTHI_PATHWAY | |
| 20 | BIOCARTA_PAR1_PATHWAY | |

**Table S6: Most Differentially Ranked Genes**

| **Rank** | **Muscle Genes** | **Muscle DOM** | **Lung Genes** | **Lung DOM** |
| --- | --- | --- | --- | --- |
| 1 | IFNA17 | 3.7 | MEFV | 3.59 |
| 2 | CCR5 | 3.68 | PRKCD | 3.19 |
| 3 | TG | 3.66 | HIST2H2AA3 | 3.16 |
| 4 | DPF2 | 3.58 | HIST1H4C | 3.15 |
| 5 | XRCC3 | 3.49 | PLA2G4D | 3.12 |
| 6 | CYP4F2 | 3.42 | DCTD | 3.02 |
| 7 | PIN1 | 3.41 | VASP | 2.97 |
| 8 | HK2 | 3.39 | FOS | 2.94 |
| 9 | RPS4Y1 | 3.38 | NGFR | 2.93 |
| 10 | PDHA2 | 3.32 | CYP2B6 | 2.88 |
| 11 | FUT5 | 3.27 | TWIST1 | 2.88 |
| 12 | TAF12 | 3.26 | CBR1 | 2.88 |
| 13 | G6PC | 3.22 | CDC7 | 2.87 |
| 14 | NUMBL | 3.21 | ALDH1B1 | 2.83 |
| 15 | CA9 | 3.13 | ND6 | 2.83 |
| 16 | GNAO1 | 3.1 | BCKDHA | 2.82 |
| 17 | HSD17B8 | 3.09 | ID1 | 2.82 |
| 18 | ABCG4 | 3.05 | CYBA | 2.81 |
| 19 | GAB2 | 2.99 | CLDN3 | 2.79 |
| 20 | PDE2A | 2.97 | PPAP2C | 2.76 |
| 21 | CBR1 | 2.96 | UBE2M | 2.75 |
| 22 | HNF1B | 2.96 | CCNA2 | 2.75 |
| 23 | ATP1A1 | 2.96 | OGDHL | 2.74 |
| 24 | PRKG2 | 2.96 | LSM7 | 2.72 |
| 25 | TOMM40L | 2.94 | PLOD3 | 2.65 |
| 26 | DYNLL2 | 2.93 | TRMT11 | 2.65 |
| 27 | CLDN20 | 2.9 | SGPP2 | 2.64 |
| 28 | CYP27A1 | 2.89 | BHLHE40 | 2.62 |
| 29 | MC1R | 2.85 | ITGB1 | 2.61 |
| 30 | CD1A | 2.85 | LY96 | 2.6 |
| 31 | ULBP1 | 2.84 | CLOCK | 2.59 |
| 32 | LRRC4C | 2.84 | ENPP7 | 2.59 |
| 33 | WNT2 | 2.83 | PLCB3 | 2.59 |
| 34 | CCL27 | 2.82 | TP53 | 2.58 |
| 35 | HMOX1 | 2.81 | EPHX2 | 2.56 |
| 36 | CXCL2 | 2.79 | UBE2S | 2.51 |
| 37 | ABCB4 | 2.78 | ACAT1 | 2.51 |
| 38 | HIST1H2AI | 2.77 | NEIL2 | 2.5 |
| 39 | SERPINA1 | 2.77 | TECR | 2.48 |
| 40 | POLA1 | 2.77 | HPRT1 | 2.48 |
| 41 | POLR3A | 2.76 | MIF | 2.46 |
| 42 | ID3 | 2.76 | COPA | 2.45 |
| 43 | BDKRB1 | 2.75 | ATP6V0A4 | 2.44 |
| 44 | GCNT4 | 2.74 | DET1 | 2.43 |
| 45 | HIST1H2AJ | 2.74 | OLR1 | 2.42 |
| 46 | NAGK | 2.73 | FUT1 | 2.42 |
| 47 | MAFG | 2.72 | ERBB2 | 2.41 |
| 48 | TAS2R9 | 2.72 | HIST1H2AB | 2.39 |
| 49 | CACNA2D3 | 2.7 | SF3B14 | 2.38 |
| 50 | ETNK2 | 2.69 | FCGR3B | 2.38 |
| 51 | EFNA1 | 2.69 | HIST1H2AE | 2.38 |
| 52 | CA1 | 2.69 | SH2B2 | 2.37 |
| 53 | GTF2H4 | 2.68 | FANCE | 2.37 |
| 54 | XDH | 2.68 | RPA1 | 2.37 |
| 55 | EXOSC9 | 2.68 | CYP1A1 | 2.34 |
| 56 | DEGS2 | 2.66 | ACO2 | 2.34 |
| 57 | LAMC3 | 2.66 | ODC1 | 2.34 |
| 58 | NAIP | 2.66 | NTF4 | 2.33 |
| 59 | PPP1R14A | 2.65 | SPP1 | 2.33 |
| 60 | MANBA | 2.65 | FCGR2A | 2.33 |
| 61 | CLDN9 | 2.64 | PAQR7 | 2.32 |
| 62 | ST3GAL6 | 2.63 | GTF2H2 | 2.32 |
| 63 | LDHAL6A | 2.63 | ATG4D | 2.3 |
| 64 | PRODH2 | 2.63 | PPAP2B | 2.3 |
| 65 | PSMB8 | 2.62 | GNAZ | 2.3 |
| 66 | CD5 | 2.62 | MICA | 2.29 |
| 67 | CASP5 | 2.6 | LRRK2 | 2.29 |
| 68 | GNPTG | 2.59 | EHHADH | 2.28 |
| 69 | HAO2 | 2.59 | SDHD | 2.27 |
| 70 | HSPG2 | 2.58 | WASF1 | 2.27 |
| 71 | CTSA | 2.58 | CLTCL1 | 2.27 |
| 72 | KIR2DL5A | 2.58 | LMNB1 | 2.27 |
| 73 | IFNA16 | 2.57 | XRN2 | 2.27 |
| 74 | IFNA6 | 2.57 | WNT5A | 2.26 |
| 75 | PRPF6 | 2.56 | DLG4 | 2.26 |
| 76 | PLD2 | 2.55 | RFXANK | 2.26 |
| 77 | TNFRSF1B | 2.53 | AXIN1 | 2.26 |
| 78 | EPHX2 | 2.53 | CD14 | 2.26 |
| 79 | SMPD2 | 2.52 | EME1 | 2.24 |
| 80 | PSAPL1 | 2.52 | ULBP3 | 2.24 |
| 81 | UPRT | 2.52 | NPR2 | 2.24 |
| 82 | AASS | 2.5 | PGD | 2.23 |
| 83 | GADD45A | 2.5 | MPG | 2.23 |
| 84 | ENPP3 | 2.5 | TFG | 2.22 |
| 85 | PTPN7 | 2.5 | UNG | 2.21 |
| 86 | INHBC | 2.49 | CARD8 | 2.21 |
| 87 | IFNA14 | 2.48 | PLXNB2 | 2.21 |
| 88 | TNFRSF13B | 2.48 | ALAS1 | 2.2 |
| 89 | ACACA | 2.47 | TAF5 | 2.19 |
| 90 | GPAT2 | 2.46 | SPN | 2.19 |
| 91 | RPRM | 2.45 | XRCC3 | 2.19 |
| 92 | NCR2 | 2.45 | XYLT1 | 2.18 |
| 93 | FANCE | 2.45 | AP4M1 | 2.18 |
| 94 | SLC2A1 | 2.44 | MAP3K9 | 2.18 |
| 95 | ITPA | 2.44 | DYNC2H1 | 2.18 |
| 96 | GCH1 | 2.43 | ICAM2 | 2.18 |
| 97 | PYGB | 2.43 | IGLL1 | 2.18 |
| 98 | EPHX1 | 2.42 | ASL | 2.18 |
| 99 | ST6GALNAC6 | 2.42 | G6PD | 2.17 |
| 100 | GPX2 | 2.41 | PRKACG | 2.17 |

**Table S7: Loadings for PC2**

|  | **PC2 Largest Positive Loadings (Adenoma)** | **Loading** | **PC2 Largest Negative Loadings (IBD)** | **Loading** |
| --- | --- | --- | --- | --- |
| 1 | BIOCARTA_AKAP95_PATHWAY | 0.126 | KEGG_PANTOTHENATE_AND_COA_BIOSYNTHESIS | -0.122 |
| 2 | BIOCARTA_NEUROTRANSMITTERS_PATHWAY | 0.116 | BIOCARTA_41BB_PATHWAY | -0.114 |
| 3 | BIOCARTA_RNA_PATHWAY | 0.112 | BIOCARTA_ARENRF2_PATHWAY | -0.107 |
| 4 | KEGG_MISMATCH_REPAIR | 0.109 | KEGG_TRYPTOPHAN_METABOLISM | -0.107 |
| 5 | KEGG_PROPANOATE_METABOLISM | 0.108 | BIOCARTA_NKCELLS_PATHWAY | -0.105 |
| 6 | KEGG_NUCLEOTIDE_EXCISION_REPAIR | 0.108 | BIOCARTA_TOLL_PATHWAY | -0.105 |
| 7 | BIOCARTA_RB_PATHWAY | 0.106 | BIOCARTA_CDC42RAC_PATHWAY | -0.99 |
| 8 | BIOCARTA_G2_PATHWAY | 0.104 | KEGG_PATHOGENIC_ESCHERICHIA_COLI_INFECTION | -0.98 |
| 9 | KEGG_DNA_REPLICATION | 0.104 | BIOCARTA_MEF2D_PATHWAY | -0.98 |
| 10 | BIOCARTA_CELLCYCLE_PATHWAY | 0.101 | BIOCARTA_D4GDI_PATHWAY | -0.98 |
| 11 | BIOCARTA_ATRBRCA_PATHWAY | 0.098 | KEGG_B_CELL_RECEPTOR_SIGNALING_PATHWAY | -0.097 |
| 12 | BIOCARTA_MCM_PATHWAY | 0.098 | BIOCARTA_TRKA_PATHWAY | -0.096 |
| 13 | BIOCARTA_TEL_PATHWAY | 0.097 | BIOCARTA_MONOCYTE_PATHWAY | -0.091 |
| 14 | KEGG_RIBOFLAVIN_METABOLISM | 0.097 | KEGG_CIRCADIAN_RHYTHM_MAMMAL | -0.09 |
| 15 | KEGG_HOMOLOGOUS_RECOMBINATION | 0.095 | KEGG_FOLATE_BIOSYNTHESIS | -0.089 |
| 16 | BIOCARTA_EIF2_PATHWAY | 0.095 | KEGG_VALINE_LEUCINE_AND_ISOLEUCINE_BIOSYNTHESIS | -0.089 |
| 17 | BIOCARTA_RACCYCD_PATHWAY | 0.093 | BIOCARTA_GABA_PATHWAY? | -0.088 |
| 18 | BIOCARTA_NKT_PATHWAY | 0.091 | KEGG_LINOLEIC_ACID_METABOLISM | -0.087 |
| 19 | BIOCARTA_SARS_PATHWAY | 0.09 | KEGG_VITCB_PATHWAY | -0.086 |
| 20 | BIOCARTA_ERK_PATHWAY | 0.088 | KEGG_FC_EPSILON_RI_SIGNALING_PATHWAY | -0.085 |

Yellow: Immune response

Light blue: DNA damage and stress response

Green: Metabolism

Red: Signaling

Pink: Cell cycle regulation

**Table S8: Loadings for PC3**

|  | **PC3 Largest Positive Loadings (Adenoma)** | **Loading** | **PC3 Largest Negative Loadings (CRC)** | **Loading** |
| --- | --- | --- | --- | --- |
| 1 | BIOCARTA_TNFR2_PATHWAY | 0.181 | BIOCARTA_STEM_PATHWAY | -0.149 |
| 2 | BIOCARTA_CD40_PATHWAY | 0.149 | BIOCARTA_GRANULOCYTES_PATHWAY | -0.143 |
| 3 | BIOCARTA_EGFR_SMRTE_PATHWAY | 0.12 | BIOCARTA_FREE_PATHWAY | -0.135 |
| 4 | BIOCARTA_FEEDER_PATHWAY | 0.117 | BIOCARTA_INFLAM_PATHWAY | -0.125 |
| 5 | BIOCARTA_P53HYPOXIA_PATHWAY | 0.11 | BIOCARTA_IL17_PATHWAY | -0.122 |
| 6 | KEGG_NITROGEN_METABOLISM | 0.108 | BIOCARTA_ERYTH_PATHWAY | -0.118 |
| 7 | KEGG_GLYCOSPHINGOLIPID_BIOSYNTHESIS_LACTO_AND_NEOLACTO_SERIES | 0.107 | BIOCARTA_P27_PATHWAY | -0.115 |
| 8 | KEGG_O_GLYCAN_BIOSYNTHESIS | 0.103 | BIOCARTA_SET_PATHWAY | -0.114 |
| 9 | BIOCARTA_CBL_PATHWAY | 0.103 | BIOCARTA_LYM_PATHWAY? | -0.109 |
| 10 | BIOCARTA_UCALPAIN_PATHWAY | 0.101 | BIOCARTA_IL1R_PATHWAY | -0.109 |
| 11 | BIOCARTA_GABA_PATHWAY | 0.096 | KEGG_TAURINE_AND_HYPOTAURINE_METABOLISM | -0.101 |
| 12 | KEGG_LINOLEIC_ACID_METABOLISM | 0.096 | BIOCARTA_EPONFKB_PATHWAY | -0.1 |
| 13 | BIOCARTA_RELA_PATHWAY | 0.089 | BIOCARTA_CTL_PATHWAY | -0.099 |
| 14 | BIOCARTA_MTA3_PATHWAY | 0.088 | KEGG_PROTEIN_EXPORT | -0.097 |
| 15 | BIOCARTA_ARAP_PATHWAY | 0.087 | BIOCARTA_LAIR_PATHWAY | -0.093 |
| 16 | BIOCARTA_HDAC_PATHWAY | 0.085 | BIOCARTA_D4GDI_PATHWAY | -0.092 |
| 17 | BIOCARTA_SODD_PATHWAY | 0.083 | BIOCARTA_VEGF_PATHWAY | -0.091 |
| 18 | KEGG_PROXIMAL_TUBULE_BICARBONATE_RECLAMATION | 0.082 | BIOCARTA_PLATELETAPP_PATHWAY | -0.089 |
| 19 | KEGG_TASTE_TRANSDUCTION | 0.082 | KEGG_GRAFT_VERSUS_HOST_DISEASE | -0.089 |
| 20 | KEGG_FOLATE_BIOSYNTHESIS | 0.08 | KEGG_ONE_CARBON_POOL_BY_FOLATE | -0.088 |

Yellow: Immune response

Light blue: DNA damage and stress response

Green: Metabolism

Red: Signaling

Pink: Cell cycle regulation

**Figure S1: Example Binary** **
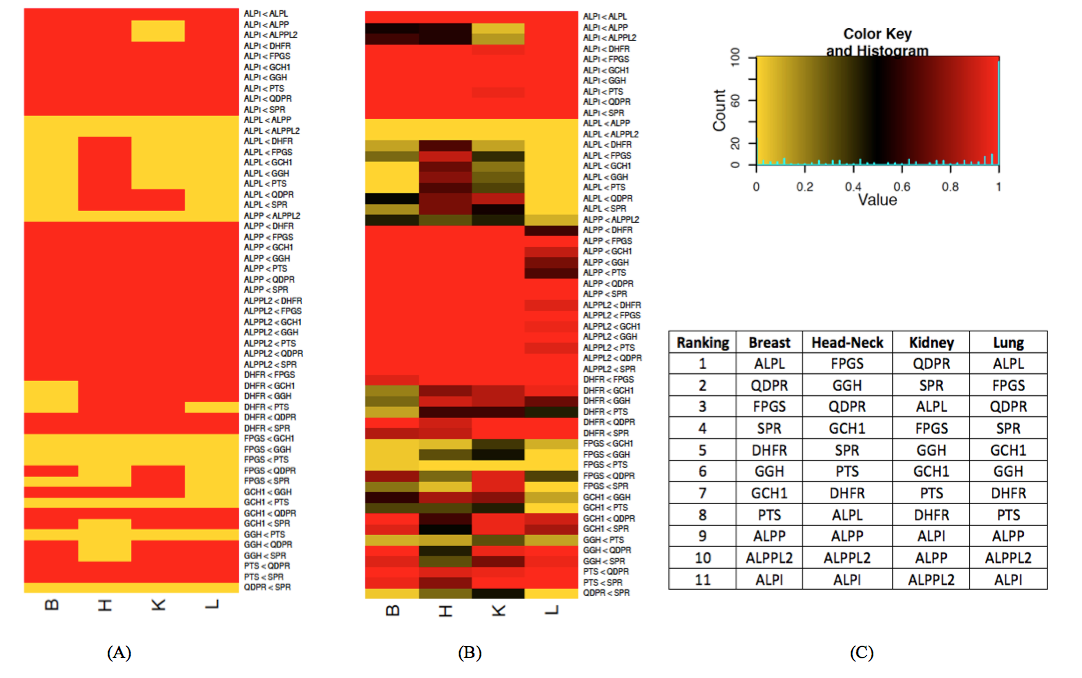
Template and Probability Template for Four Tissues**

**Figure S1:** **Example Pathway** **Template and Probability Template for Four Tissues**

The binary templates (A) and probability templates (B) for the “Folate Biosynthesis” KEGG pathway for each of breast, head-neck, kidney and lung (denoted B, H, K and L respectively) tissues. This pathway consists of 11 genes. The corresponding 55 gene pairs are shown in the heatmaps. In both DIRAC and GRAPE classification, samples are assigned to the tissue for which the template most closely matches the ranking of the genes in the sample. In DIRAC the distance is simply the number of inversions. In GRAPE each inversion is weighted by a function of the probability template. The DIRAC-estimated accuracy is 0.82 and the GRAPE-estimated accuracy is 0.90 for this pathway. Accuracies estimated for this pathway by random forest and SVM are 0.96 and 0.93, respectively. Although many other pathways achieved higher classification accuracies, as seen in Figure 1, this pathway was chosen because the small number of genes enables easy visualization. The pairwise orderings from the binary template are converted to full ordering sequences for each tissue type (C).

**Figure S2: Mean Classification Accuracy Across Bins of Different Pathway Lengths**


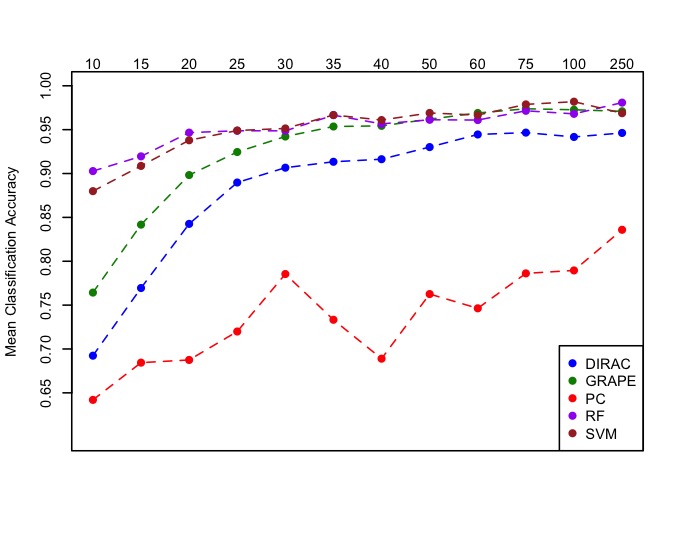


**Figure S2: Mean Classification Accuracy Across Bins of Different Pathway Lengths**

The full set of KEGG and BioCarta pathways were partitioned into 12 bins according to pathway size. The bin labels (top) indicate the largest pathway lengths that are included in each bin (e.g. bin 12 consists of all pathways with between 101 and 250 genes). For each of the five methods, the average classification accuracy was calculated over each bin for four TCGA tissue types. The results show that the classification performances of GRAPE and DIRAC are more sensitive to pathway length than those of RF and SVM.

**Figure S3: Classification Across 4 Agilent Datasets, 2 Lung 2 Muscle**


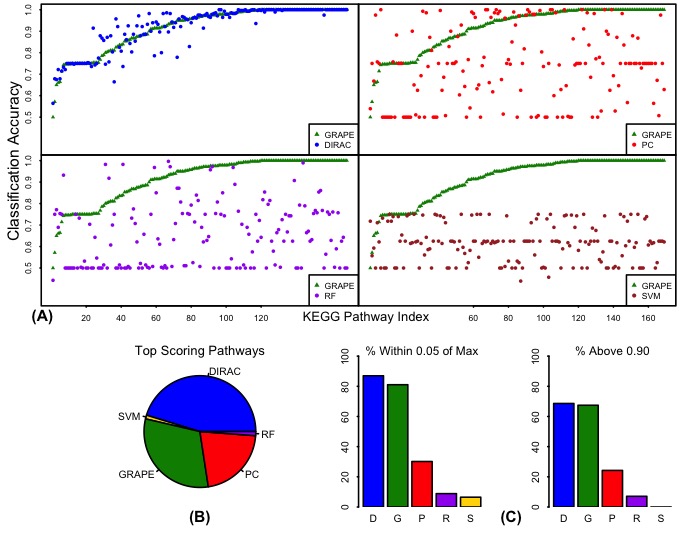


**Figure S3: Classification Across 4 Agilent Datasets, 2 Lung 2 Muscle**

Classification performance using two separate datasets for each of two healthy tissues: muscle and lung. The four datasets were generated using the same Agilent microarray (GPL6480). Muscle datasets were GSE23697 and GSE42507, lung datasets were GSE15197 and GSE40588. There are four possible ways to split the datasets such that each tissue type is contained in each of the training and testing sets. The score for each pathway is the average of all four splits. B) Pie chart indicating proportion of pathways for which each method had largest accuracy: 45%, 31%, 21%, 1% and 1% for DIRAC, GRAPE, PC, RF and SVM, respectively. Fractions were used to account for ties. Accuracy within .001 of max counted as max. C) Proportion of pathways for each method that achieve classification accuracy within 0.05 of the best performance (left) and proportion that achieve at least 0.90 classification accuracy (right).

**Figure S4: Classification Across 3 Affymetrix and 3 Agilent Datasets**


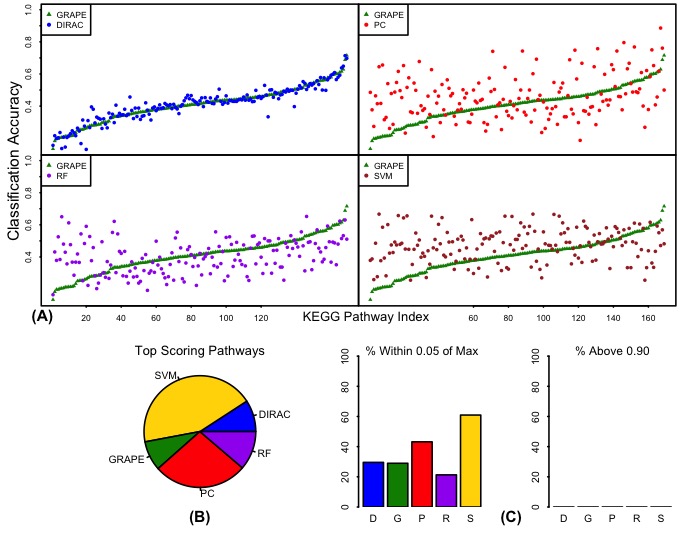


**Figure S4: Classification Across 3 Affymetrix and 3 Agilent Datasets**

Classification performance using two separate datasets for each of three healthy tissues: muscle, lung and colon. For each tissue type one Affymetrix dataset was chose and one Agilent dataset was chosen. The muscle datasets were GSE47881 (Affymetrix) and GSE42507 (Agilent). The lung datasets were GSE4302 (Affymetrix) and GSE40588 (Agilent). The colon datasets were GSE4183 (Affymetrix) and GSE41657 (Agilent). To ensure balance, for each of the six datasets, a randomly selected subset of 23 samples was chosen and the rest of the samples were discarded from the analysis. There are eight possible ways to split the datasets such that each tissue type is contained in each of the training and testing sets. The score for each pathway is the average of all eight splits. B) Pie chart indicating proportion of pathways for which each method had largest accuracy: 9%, 9%, 27%, 11% and 44% for DIRAC, GRAPE, PC, RF and SVM, respectively. Fractions were used to account for ties. Accuracy within .001 of max counted as max. C) Proportion of pathways for each method that achieve classification accuracy within 0.05 of the best performance (left) and proportion that achieve at least 0.90 classification accuracy (right).

**Figure S5: Distribution of Differentially Ranked Pairs Across Genes**

**
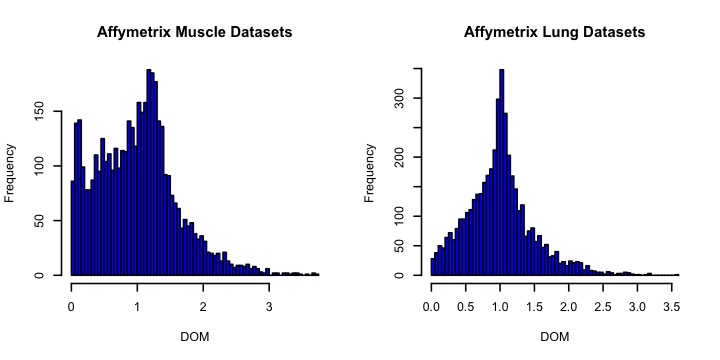
**

**(A)**

**
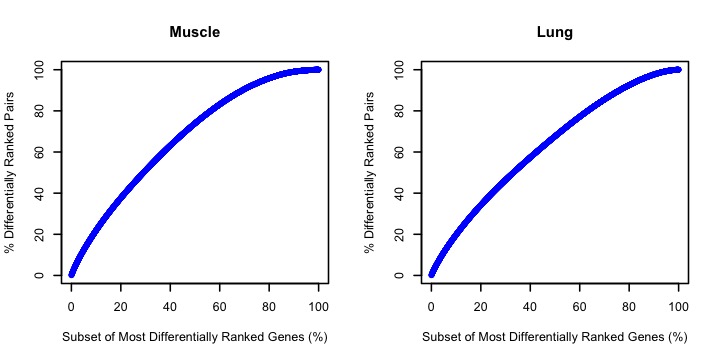
**

**(B)**

**Figure S5: Distribution of Differentially Ranked Pairs Across Genes**

(A) Distribution of Differential Ordering Measures (DOM) of 4413 KEGG and BioCarta genes. (B) Shown is the contribution of the most differentially ordered genes to the entire set of significantly differentially ranked pairs.

**Figure S6: GRAPE vs. Combat Classification Over Multiple Datasets**


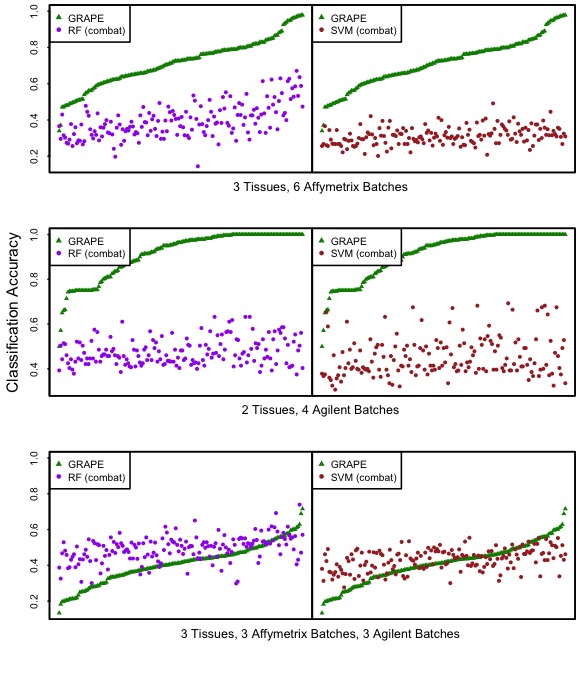


**Figure S6: GRAPE vs. Combat Classification Over Multiple Datasets**

Classification performances of GRAPE from Figures 2, S3 and S4 are compared with classification performance of RF and SVM after applying combat to remove batch effects. (Top) Combat was applied to the six Affymetrix batches that were used in Figure 2. (Middle) Combat was applied to the four Agilent batches that were used in Figure S3. (Bottom) Combat was applied to the three Affymetrix and three Agilent batches that were used in Figure S4.

**Figure S7: Similarity of GRAPE, GSEA Pathway Ranks for BRCA Subtypes**

**
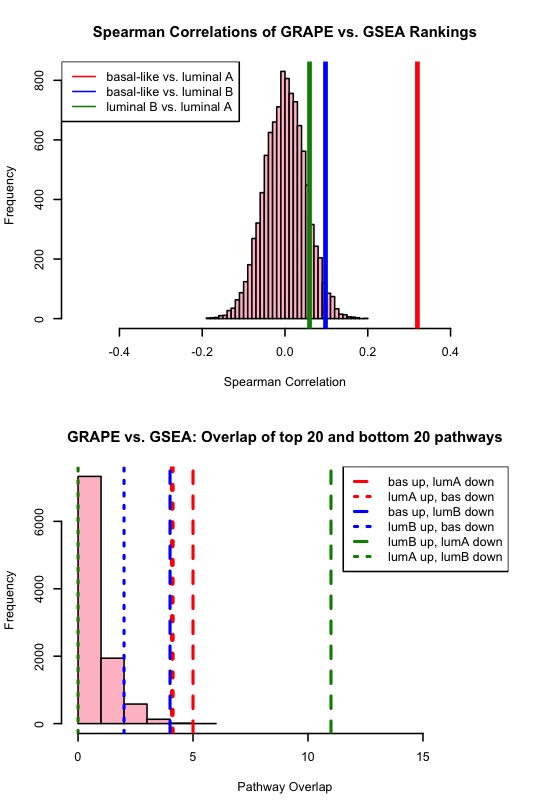
**

**Figure S7: Similarity of GRAPE, GSEA Pathway Ranks for BRCA Subtypes**

For each subtype comparison, pathways were ranked according to most significantly upregulated in class 1 to most significantly upregulated in class 2. (Top) Spearman correlations for each pair of subtypes. The pink histogram shows the null distribution from 10,000 pairs of randomly ranked pathway lists. (Bottom) Pathway overlap of the top and bottom of the GRAPE and GSEA ranked lists. The pink histogram shows the null distribution of overlapping top 20 pathways from 10,000 pairs of randomly ranked pathway lists.

**Figure S8: Volcano Plots of Differential Pathway Expression between Breast Cancer Subtypes**


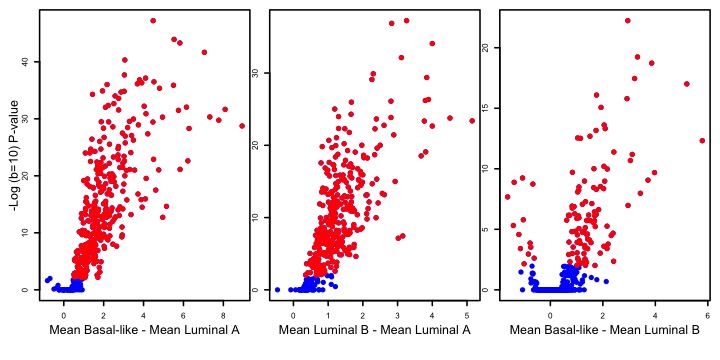


**Figure S8: Volcano Plots of Differential Pathway Expression between Breast Cancer Subtypes**

**Shown is a volcano plots of pathway score differences between pairs of breast cancer subtypes. For each panel, y-axis is negative log of Bonferroni adjusted p-values, and x-axis is the mean difference between the two indicated subtypes subtypes.**

**
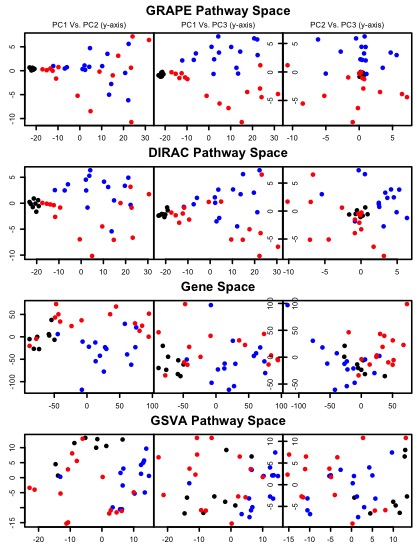
Figure S9: PC plots of three colon diseases**

**Figure S9: PC plots of three colon diseases**

Pairwise plots of first three principal components (PC) for four representations of three colonic tissue types. Normal colon in black, adenomas in blue, and colorectal cancer in red. For GRAPE, DIRAC and GSVA, pathway scores were calculated for 397 KEGG and BioCarta pathways. The gene space representation consists of the gene expression matrix after removal of genes with standard deviation below 0.01.

**Figure S10: Distribution of PC2 and PC3 Loadings**


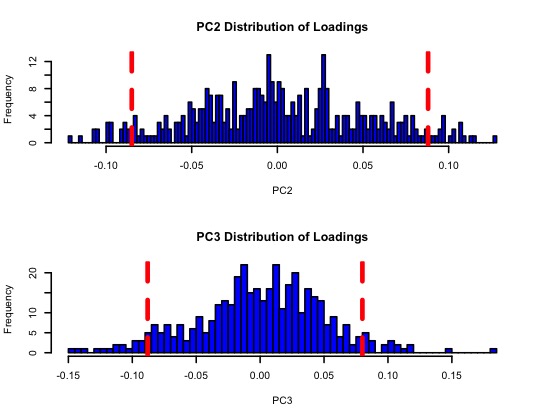


**Figure S10: Distribution of PC2 and PC3 Loadings**

Distribution of principal component loadings for all pathways for PC2 and PC3. Dashed line indicates the position of the 20^th^ most extreme pathway loading. Pathways whose loadings are outside the dashed lines are tabulated in Tables S6 and S7.

**Figure S11: Classification performance of GRAPE vs. DIRAC for different numbers of classes**

**Figure S11: Classification performance of GRAPE vs. DIRAC for different numbers of classes**

Separate classification experiments were performed using two through six classes. The data for this analysis were RNA-Seq profiles of different healthy tissues from the GTEx project [[2](#_ENREF_2)]. The two classes experiment consisted of lung and muscle tissue types. Adipose was added for the three classes experiment. Skin, heart and thyroid were sequentially added for the experiments of four, five and six classes, respectively. Fifty samples of each tissue type were used for each experiment. The pathway set for this analysis was the set of all KEGG pathways having between 20 and 100 genes. The average of four iterations of five-fold cross validation was calculated for each pathway. The green points show

the percentage of pathways for which GRAPE strictly outperformed DIRAC. The blue points show

the percentage of pathways for which DIRAC strictly outperformed GRAPE. The brown points show the percentage of pathways for which both methods achieved identical classification accuracies. The results show that the comparative performance improvement of GRAPE relative to DIRAC increases as the number of classes increases.

References

1. Markowitz, H., *PORTFOLIO SELECTION*.* The Journal of Finance, 1952. **7**(1): p. 77-91.

2. Consortium, T.G., *The Genotype-Tissue Expression (GTEx) pilot analysis: Multitissue gene regulation in humans.* Science, 2015. **348**(6235): p. 648-660.
